# Photoactivated Gallium Porphyrin Reduces *Staphylococcus aureus* Colonization on the Skin and Suppresses Its Ability to Produce Enterotoxin C and TSST-1

**DOI:** 10.1021/acs.molpharmaceut.3c00399

**Published:** 2023-09-01

**Authors:** Klaudia Szymczak, Grzegorz Szewczyk, Michał Rychłowski, Tadeusz Sarna, Lei Zhang, Mariusz Grinholc, Joanna Nakonieczna

**Affiliations:** † Laboratory of Photobiology and Molecular Diagnostics, Intercollegiate Faculty of Biotechnology, University of Gdansk and Medical University of Gdansk, Gdansk 80-307, Poland; ‡ Department of Biophysics, Faculty of Biochemistry, Biophysics and Biotechnology, Jagiellonian University, Krakow 30-387, Poland; § Laboratory of Virus Molecular Biology, Intercollegiate Faculty of Biotechnology, University of Gdansk and Medical University of Gdansk, Gdansk 80-307, Poland; ∥ Department of Biochemical Engineering, School of Chemical Engineering and Technology, Frontier Science Center for Synthetic Biology and Key Laboratory of Systems Bioengineering (MOE), Tianjin University, Tianjin 300350, China

**Keywords:** antimicrobial treatment, atopic dermatitis, photodynamic inactivation, reactive oxygen species, superantigens

## Abstract

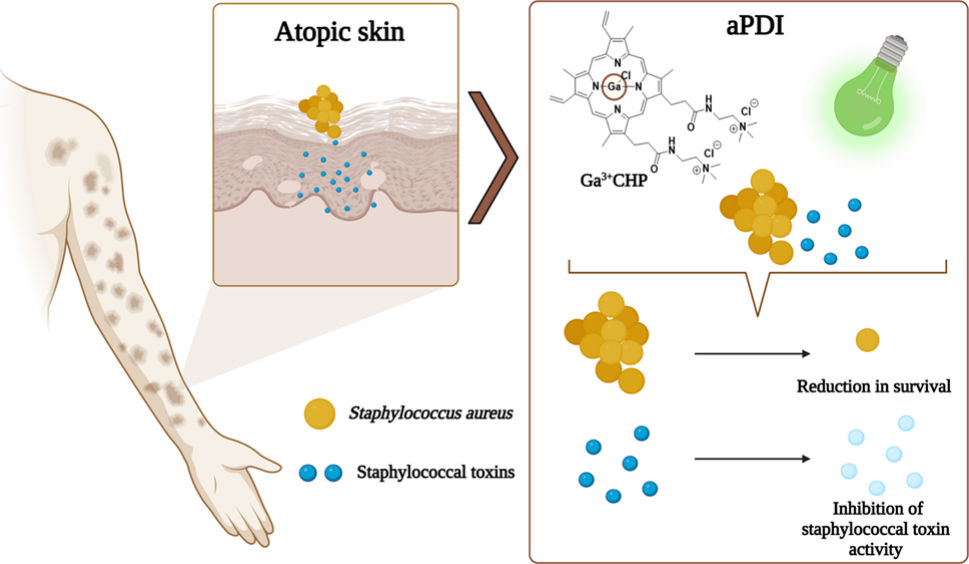

*Staphylococcus aureus* is a key pathogen in atopic dermatitis (AD) pathogenicity. Over half of AD patients are carriers of *S. aureus*. Clinical isolates derived from AD patients produce various staphylococcal enterotoxins, such as staphylococcal enterotoxin C or toxic shock syndrome toxin. The production of these virulence factors is correlated with more severe AD. In this study, we propose cationic heme-mimetic gallium porphyrin (Ga^3+^CHP), a novel gallium metalloporphyrin, as an anti-staphylococcal agent that functions through dual mechanisms: a light-dependent mechanism (antimicrobial photodynamic inactivation, aPDI) and a light-independent mechanism (suppressing iron metabolism). Ga^3+^CHP has two additive quaternary ammonium groups that increase its water solubility. Furthermore, Ga^3+^CHP is an efficient generator of singlet oxygen and can be recognized by heme-target systems such as Isd, which improves the intracellular accumulation of this compound. Ga^3+^CHP activated with green light effectively reduced the survival of clinical *S. aureus* isolates derived from AD patients (>5 log_10_ CFU/mL) and affected their enterotoxin gene expression. Additionally, there was a decrease in the biological functionality of studied toxins regarding their superantigenicity. In aPDI conditions, there was no pronounced toxicity in HaCaT keratinocytes with both normal and suppressed filaggrin gene expression, which occurs in ∼50% of AD patients. Additionally, no mutagenic activity was observed. Green light-activated gallium metalloporphyrins may be a promising chemotherapeutic to reduce *S. aureus* colonization on the skin of AD patients.

## 1 Introduction

The ESKAPE pathogens (*Enterococcus faecium*, *Staphylococcus aureus*, *Klebsiella pneumoniae*, *Acinetobacter baumannii*, *Pseudomonas aeruginosa*, and *Enterobacter species*) are the leading cause of infections throughout the world. *Staphylococcus aureus* is a Gram-positive bacterium and a key member of the ESKAPE superbugs, which are considered a dynamic group of emerging antimicrobial-resistant pathogens.^1^*S. aureus* produces a range of virulence factors, such as staphylococcal enterotoxins (SEs), that increase its virulence and pathogenicity.^2,3^ SEs such as staphylococcal enterotoxin C (SEC) or toxic shock syndrome toxin (TSST-1) are potent, nonspecific superantigens that stimulate over 50% of the T-cell pool.^4^ SEs aggravate and enhance inflammation in atopic dermatitis (AD) patients.^5^ Clinical isolates of *S. aureus* derived from AD patients are a genetically heterogeneous population in terms of the presence of superantigen genes. AD patients are a source of specific isolates that are more potent in colonizing AD skin and altering immunological responses.^6,7^

AD is a multifactorial chronic inflammatory skin disorder that affects both adults and children, and its occurrence has increased over the last decade.^3,8^ Crucial factors involved in AD development are genetic background, immune system disorders, and defects in the epidermal barrier.^9^ These factors influence the skin microbiome.^2,3^ Compared with healthy individuals, AD patients show higher colonization levels of methicillin-resistant *S. aureus* (MRSA) strains. Approximately 55% of AD patients are persistent carriers of *S. aureus*.^10^ Staphylococcal colonization might be related to changes in skin pH and low levels of ceramides and antimicrobial peptides.^5^ There is also a correlation between a mutation in the filaggrin gene (*FLG*) and increased *S. aureus* colonization on the skin of AD patients.^11^ Many cohort studies have demonstrated that 25–50% of AD patients possess a mutation in the *FLG* gene.^12^ Filaggrin is a key protein that maintains proper hydration and epidermal integrity by cross-linking keratin filaments. Lack of this protein significantly enhances allergen and microbial penetration into the skin.^13,14^

Antimicrobial AD treatment is not yet predominant due to the multifactorial nature of the disease. Antibiotic therapy remains the gold standard treatment for fighting staphylococcal infections in AD patients. However, the number of available and effective antimicrobials is shrinking due to increasing antimicrobial resistance. Antimicrobial photodynamic inactivation (aPDI) might be an alternative way to reduce *S. aureus* colonization on atopic skin. This approach is based on three components: oxygen, light at the proper wavelength, and a compound known as a photosensitizer (PS). Briefly, under light illumination, the PS is excited to its triplet state, and then two types of mechanisms can occur. In the type I mechanism, electrons are transferred between the excited PS and biomolecules to produce cytotoxic reactive oxygen species (ROS) such as the superoxide anion, hydrogen peroxide, and/or hydroxyl radicals.^15^ In the type II reaction, singlet oxygen is produced by transferring energy of the excited PS to molecular oxygen. To date, there has been no evidence of antimicrobial resistance to aPDI.^16^ Gallium metalloporphyrins (Ga^3+^MPs) are effective PSs in aPDI against *S. aureus* despite divergent multidrug responses.^17^ Ga^3+^MPs are dual-function compounds that act according to light-independent and light-dependent mechanisms, and they mimic their natural analogue—heme.^18^ On the staphylococcal membrane, there are two types of heme acquisition receptors, isd (iron-surface determinate) or hts (heme transport system), that can recognize Ga^3+^MPs in the same manner as heme, allowing Ga^3+^MPs to accumulate inside the cell.^19^ After cleavage of the porphyrin ring, gallium ions are released and inhibit iron-dependent metabolic pathways. Moreover, there are reports stating that Ga^3+^MPs could be detoxified in a manner similar to heme by the heme-regulated transporter HrtAB efflux pump.^20^ Ga^3+^MPs have photodynamic potential after illumination at the proper wavelength in the Soret or Q band area, which are the high- and low-energy parts of the porphyrin absorption spectrum, respectively.^17,20^ In addition, they have additive quaternary ammonium groups that increase their water solubility. Moreover, a previous study showed that this compound was effective in iron-blocking antibacterial therapy against Gram-positive and Gram-negative bacteria with visible light irradiation in the area of the Soret band.^21^ Our previous study reported that structural changes such as vinyl to ethyl groups in the structure of the porphyrin ring of gallium mesoporphyrin IX (Ga^3+^MPIX) did not change the recognition of the compound, although the aqueous solubility was increased and a shift in the absorbance spectrum was observed. Additionally, these changes improved the efficacy of aPDI against *S. aureus* under illumination with green light in the Q band region.^20^ Using the wavelengths nearest to the visible green light might be a crucial therapeutic strategy for treating AD due to deeper light penetration through the epidermal barrier.^22^

**Figure 1:**
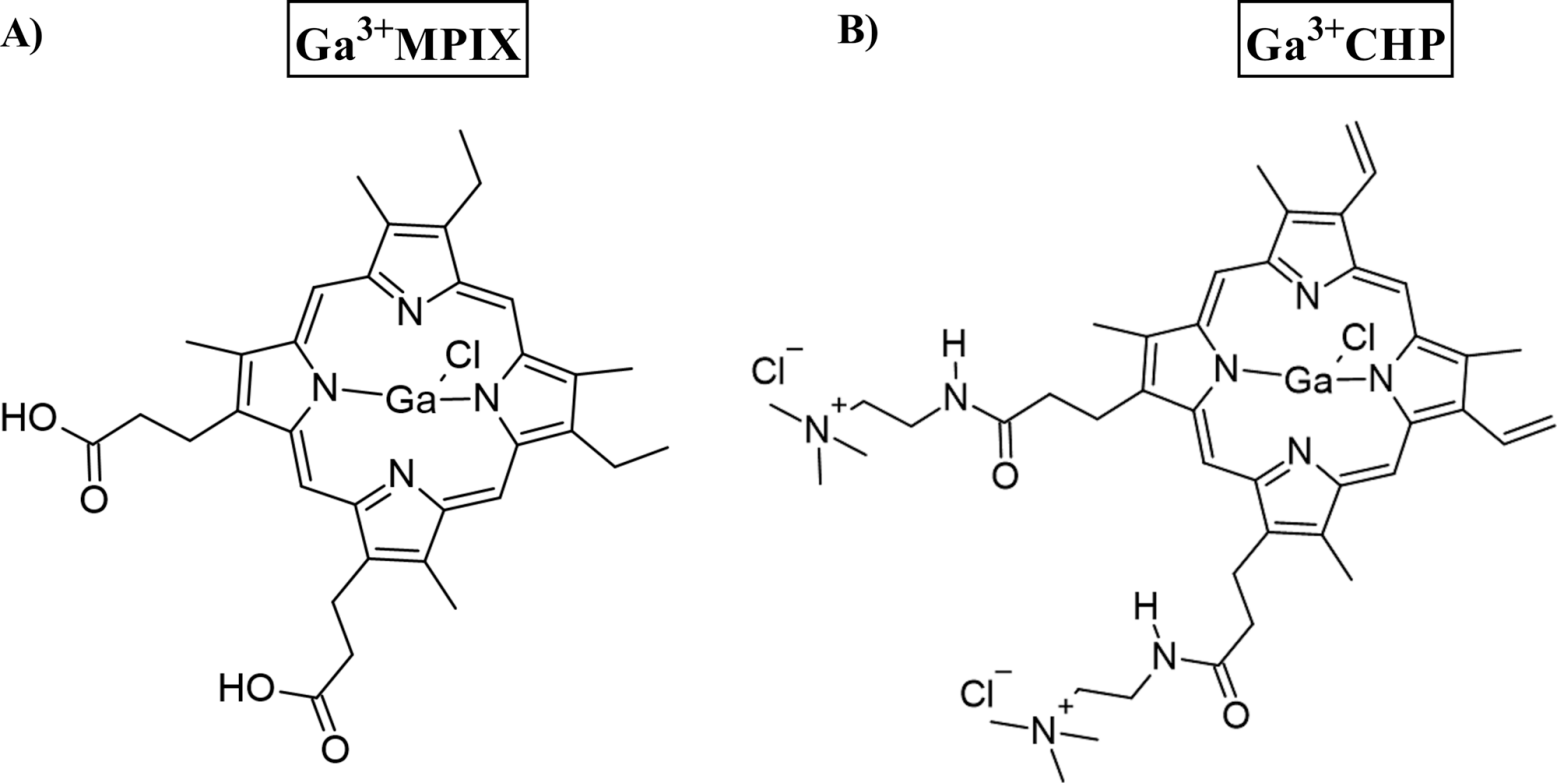
Chemical structures of Ga^3+^MPIX (A) and Ga^3+^CHP (B) drawn in ChemSketch.

In this work, the photoexcitation of Ga^3+^CHP (Figure 1) was studied under 522 nm illumination to characterize the photodynamic potential of this compound. Furthermore, we investigated whether the presence of two additive quaternary ammonium groups could affect the recognition of the compound by heme acquisition receptors and detoxification machinery. The efficacy of aPDI of both gallium compounds, Ga^3+^CHP and Ga^3+^MPIX (Figure 1), was examined against *S. aureus* clinical isolates derived from AD patients in planktonic culture and ex vivo porcine skin models. The mutagenicity and safety of the compounds in keratinocytes with divergent filaggrin expression were also investigated. Finally, the effect of aPDI with both gallium compounds on the gene expression, protein production, and biological activity of two virulence factors, SEC and TSST-1, was investigated.

## 2 Materials and Methods

### 2.1 Bacterial Strains and Growth Conditions

Bacterial strains are listed in Table 1. *S. aureus* cultures were grown in trypticase soy broth (TSB, bioMérieux, France) or TSB pretreated with Chelex-100 resin (Sigma-Aldrich, USA) in an iron-depleted medium at 37 °C on trypticase soy agar-coated plates (TSA, bioMérieux, France) with shaking (150 rpm). Erythromycin (10 μg/mL) was added to the cultivation medium of the *S. aureus* ΔIsdD mutant strain. Glycerol stocks of *E. coli* and *S. typhimurium* and the necessary growth media for mutagenicity testing were purchased from commercially available Ames Penta 2 (Xenometrix, Allschwil, Switzerland).

**Table 1:**
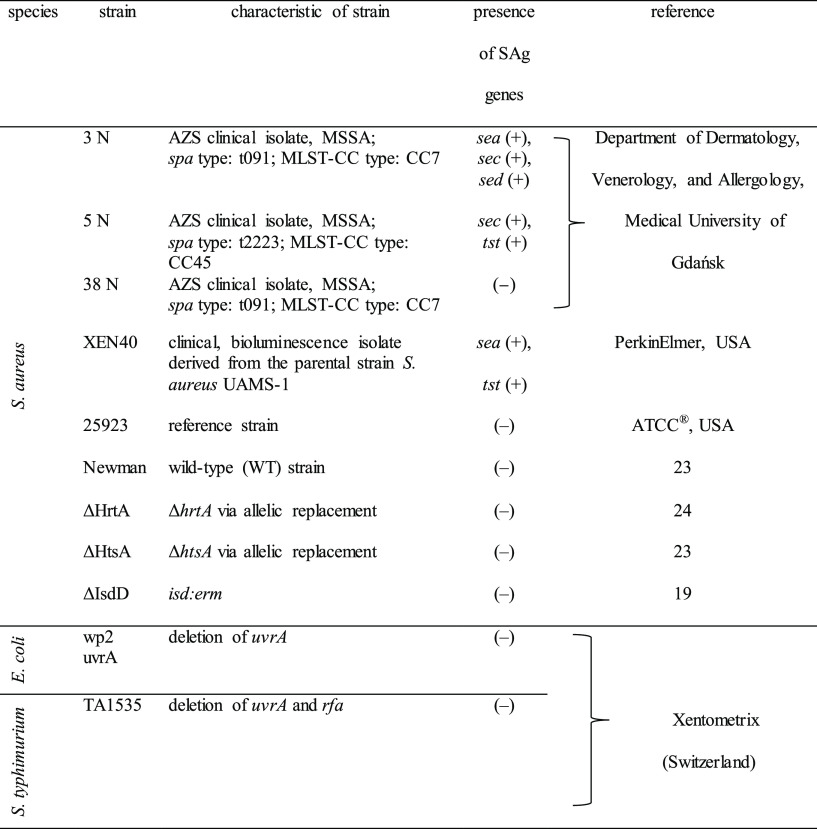
Bacterial Strains Used in This Study^a^

a Legend: MSSA—methicillin-sensitive *Staphylococcus aureus*, SAg—superantigens; *sea*, *sec*, *sed*, *tst*—staphylococcal enterotoxin A, C, D, and toxic shock syndrome toxin 1; (−)—absence of SAg genes.

### 2.2 Cell Cultures and Growth Conditions

The human immortalized keratinocyte HaCaT cell line was used in this study. Cells were either treated with empty vector (sc-108080, *FLG* ctrl) or infected with lentiviral particles containing FLG shRNA (sc-43364-V) to construct *FLG* knockdown (*FLG* sh) cells.^25^ Cells were grown in a standard humidified incubator at 37 °C in a 5% CO_2_ atmosphere in Dulbecco’s modified Eagle’s medium (DMEM) with 10% fetal bovine serum (FBS), 4.5 g/L glucose, 1 mM sodium pyruvate, 100 U/mL penicillin, 100 μg/mL streptomycin, 2 mM l-glutamine, and 1 mM nonessential amino acids (Gibco, Thermo Fisher Scientific, USA).

Human PBMCs were purified from the blood samples of healthy donors obtained from the Buffy Coat Blood Bank. Cells were harvested using Lymphoprep (Stemcell, Grenoble, France) and frozen at −80 °C until the experiment. PBMCs were cultivated in RPMI-1640 medium (Sigma-Aldrich, USA) with the addition of the supplementary cell growth additives mentioned above.

### 2.3 Chemicals

Ga^3+^ mesoporphyrin IX chloride (Ga^3+^MPIX; Frontier Scientific, USA) was prepared as previously described **(**Figure 1A).^20^ Ga^3+^CHP was synthesized, and its structure is described by Zhang et al. (Figure 1B).^21^ Five millimolar stocks of Ga^3+^CHP were prepared in Milli-Q water and kept in the dark at room temperature. Heme (Sigma-Aldrich, USA) was dissolved in a 0.1 M NaOH solution and kept in the dark at 4 °C.

### 2.4 Light Source

**Figure 2:**
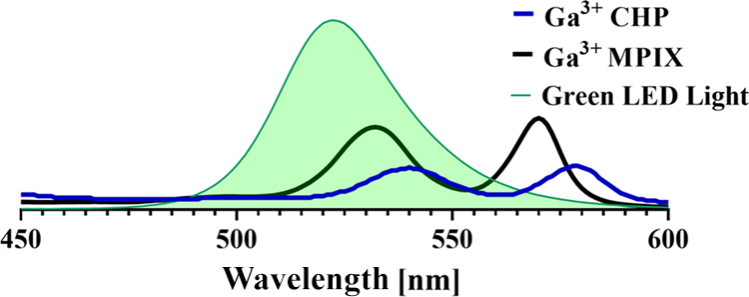
Absorbance spectra of Ga^3+^CHP and Ga^3+^MPIX [10 μM] with the emission spectrum of a green light source used in this study (λ_max_ = 522 nm, FWHM = 34 nm).

In this study, we used a light-emitting diode (LED) light source emitting green light (λ_max_ = 522 nm, irradiance = 10.6 mW/cm^2^, FWHM = 34 nm) (Cezos, Poland) (Figure 2). The irradiation time ranged from 2.5 to 50 min. During irradiation, no heat was generated (Figure S1).

### 2.5 Characterization of the Photodynamic Properties of Ga^3+^CHP

The quantum yield of Ga^3+^CHP-mediated singlet oxygen photogeneration in phosphate-buffered D_2_O was determined by comparing the initial intensities of 1270 nm phosphorescence induced by photoexcitation of both the standard rose bengal (RB) (for which the established quantum yield of singlet oxygen generation is 0.75) and the experimental photosensitizer Ga^3+^CHP with 540 nm laser pulses of increasing energies using neutral density filters. Time-resolved singlet oxygen phosphorescence induced by excitation of solutions of Ga^3+^CHP or RB, adjusted to the same absorbance at 540 nm, with nanosecond laser pulses generated by an integrated DSSNd:YAG laser system equipped with a narrow bandwidth optical parametric oscillator (NT242-1k-SH/SFG; Ekspla, Vilnius, Lithuania), was detected by a photomultiplier module H10330-45 working in the photon counting mode (Hamamatsu Photonics K. K., Hamamatsu City, Japan), equipped with a 1100 nm cut-off filter and additional dichroic narrow-band filters NBP, selectable from the spectral range 1150–1355 nm (NDC Infrared Engineering Ltd., Bates Road, Maldon, Essex, UK). Data were collected using a computer-mounted PCI-board multichannel scaler (NanoHarp 250; PicoQuant GmbH, Berlin, Germany). Data analysis, including first-order luminescence decay fitted by the Levenberg–Marquardt algorithm, was performed by custom-written software.

Electron paramagnetic resonance (EPR) spin trapping was carried out using 100 mM 5,5-dimethyl-1-pyrroline *N*-oxide (DMPO) (Dojindo Kumamoto, Japan) as a spin trap. Samples containing DMPO and approximately 0.1 mM PSs in 75% dimethylsulfoxide (DMSO) with an adjusted neutral pH were placed in 0.3 mm-thick quartz EPR flat cells and irradiated in situ in a resonant cavity with 540 nm green LED light. The EPR measurements were carried out using a Bruker-EMX AA spectrometer (Bruker BioSpin, Germany) with the following apparatus settings: 10.6 mW microwave power, 0.05 mT modulation amplitude, 332.4 mT center field, 8 mT scan field, and 84 s scan time. Simulations of EPR spectra were performed with the EasySpin toolbox for MATLAB.

### 2.6 Antimicrobial aPDI

Photoinactivation experiments were performed as previously described.^20^ Briefly, *S. aureus* was grown in either full TSB medium or TSB pretreated with Chelex-100 resin to chelate iron ions (Sigma-Aldrich, USA) for 16–20 h. Cultures were diluted to 10^7^ CFU/mL (0.5 MacFarland units), and then 90 μL of bacterial aliquots was transferred to a 96-well plate with the addition of 10 μL of either pure medium or PS (Ga^3+^MPIX or Ga^3+^CHP). For the heme competition assay, Ga^3+^CHP was mixed with heme at a ratio of 1:1 (v:v) at different concentration ratios, and then the aPDI protocol was followed. The aPDI samples were incubated at 37 °C with shaking in the dark for 10 min and illuminated with the light source (Table 2). Serial dilutions of aliquots were prepared and plated on TSA plates to calculate the colony-forming units (CFU/mL).

**Table 2:** Light Doses and Corresponding Irradiation Times Used in This Study

light dose [J/cm^2^]	irradiation time [min]
1.59	2.5
3.18	5
6.4	10
12.72	20
19.08	30
31.8	50

### 2.7 Accumulation of PS

The intracellular accumulation of each photosensitizer was determined according to our previously published protocols.^20,26^ Both compounds (1–10 μM) were added separately to bacterial aliquots to produce a final volume of 800 μL. In the heme competition assay, Ga^3+^CHP was mixed with heme in a 1:1 volume ratio (v/v) at different concentration ratios [μM:μM]. Bacterial suspensions were incubated for 10 min at 37 °C in the dark with shaking. Ten microliters of bacterial suspensions was then collected for a serial dilution to count CFU/mL. The cells were then centrifuged and washed twice with PBS. Cells were resuspended in lysis buffer (0.1 M NaOH/1% SDS) and kept for 24 h at room temperature. The fluorescence intensity of each sample was measured with an EnVision Multilabel Plate Reader (PerkinElmer, USA) at the following emission/excitation wavelengths: Ga^3+^MPIX at 406/573 nm and Ga^3+^CHP at 406/582 nm. Accumulation calculations for each PS were made from a compound calibration curve prepared in the lysis solution. The uptake values are presented as PS molecules accumulated per cell based on the previously shown formula.^26^ The molecular weight of Ga^3+^CHP was calculated to be 907.08 g/mol and that of Ga^3+^MPIX was calculated to be 669.85 g/mol.

### 2.8 aPDI in an Ex Vivo Porcine Skin Model

Ex vivo porcine skin was collected and cut into 2 × 2 cm skin grafts. They were then treated twice with 70% ethanol for 15 min, followed by PBS washing. To enhance skin decontamination before the procedure, grafts were treated with UV radiation for 15 min on each side. The grafts were then plated on a HEPES agar solid medium (10 mM HEPES, 136 mM NaCl, 4 mM KCl, 10 mM glucose, 1% agar). Overnight cultures of the bioluminescence strain of *S. aureus* Xen40 were diluted to a 0.5 MacFarland standard, and 100 μL of the bacterial suspensions was inoculated on the grafts. Bacteria were incubated at 37 °C for 24 h. The bioluminescent signal of each graft was measured by ChemiDoc XRS+ (Bio-Rad, USA) and referred to as the “before” measurement. Then, 200 μL of a 10 μM Ga^3+^CHP solution or sterile Milli-Q water was placed on the infected skin and incubated in the dark for 10 min. The skin was then exposed to green light or left in the dark. The bioluminescence signal was measured immediately after each treatment, referred to as the “after” measurement. The bioluminescence signal for each treatment at the appropriate time point was calculated using ImageJ software. The change in bioluminescent signal was measured for each condition the experiment was independently repeated in triplicate.

### 2.9 Prokaryotic Mutagenicity

The mutagenicity analysis was performed using a commercially available Ames Penta 2 kit (Xenometrix, Allschwil, Switzerland), and all steps followed the manufacturer’s protocol. The day before the experiment, three independent biological cultures of each indicator strain of *Escherichia coli* uvrA or *Salmonella typhimurium* TA1535 were prepared. After 14 h of incubation at 37 °C with shaking, the cultures were diluted in exposure medium and treated with Ga^3+^MPIX or Ga^3+^CHP. The cultures were allowed to incubate in the dark for 10 min and then irradiated with green light at the proper dose. For positive controls, cultures were treated with mutagenic chemicals such as *N*4-aminocytidine (N^4^-ACT) for *S. typhimurium* TA1535 and 4-nitroquinoline-*N*-oxide (4-NQO) for *E. coli* uvrA. Cells incubated without a compound and without light exposure were used as negative controls. All treatments were incubated for 90 min after mutagen or aPDI treatment. Exposure medium was then added to all samples, and 50 μL of each sample was aliquoted into 384-well plates. All microplates were covered with sterile foil and incubated for 48 h at 37 °C. The number of revertants after each treatment was counted following the incubation period. This experiment was performed with three independent biological replicates that each had three technical replicates of each treatment group.

### 2.10 Photo- and Cytotoxicity Assays on Human Keratinocytes

HaCat cells with silenced expression of *FLG* (*FLG* sh) and normal *FLG* gene expression (*FLG* ctrl) were tested for photo- and cytotoxicity using the MTT assay and cell growth dynamics using the xCELLigence real-time cell analyzer (RTCA) device (ACEA Biosciences Inc., USA). In the MTT assay, cells were seeded the day before the experiment at a density of 1 × 10^4^ cells per well in 96-well plates. Cells were divided into two plates for light treatment and dark control. Cells were grown in a standard humidified incubator at 37 °C in a 5% CO_2_ atmosphere in DMEM. Ga^3+^MPIX or Ga^3+^CHP was added to the cells to a final concentration of 10 μM and then incubated for 10 min at 37 °C in the dark. After incubation, the medium was changed to fresh PS-free DMEM. Next, cells were irradiated with light at 522 nm with established doses for each PS, 31.9 J/cm^2^ for Ga^3+^MPIX, and 1.59 J/cm^2^ for Ga^3+^CHP. MTT reagent was added to cells 24 h posttreatment, and after 4 h of incubation, the cells were lysed, and the absorbance of the released formazan was measured with a plate reader at 550 nm.

For real-time analysis of cell growth dynamics, each cell line was seeded the day before treatment in seven technical replicates for each condition at a density of 1 × 10^4^ per well on an E-plate (ACEA Biosciences Inc., USA). Cells were grown in a standard humidified incubator at 37 °C and a 5% CO_2_ atmosphere in DMEM on the xCELLigence device. When the cell index (CI) was in the range of 1.5–2.0, the cells were treated with aPDI. The appropriate photosensitizer was added to the cells at a concentration of 10 μM and incubated for 10 min in the dark at 37 °C. The medium was then changed to PS-free DMEM. Test cells were exposed to green light either at 31.9 J/cm^2^ for Ga^3+^MPIX or at 1.59 J/cm^2^ for Ga^3+^CHP, while control cells (treated with PS alone) were kept in the dark outside of the incubator for the same time as irradiation. After treatment, the plates were returned to the xCELLigence instrument, and the CI was measured every 10 min. Experiments were carried out until a plateau phase was reached.

### 2.11 qRT-PCR Gene Expression Analysis

RNA isolation and purification, reverse transcription, and qPCR were performed according to previously published data.^27^ Briefly, RNA was isolated and purified from the *S. aureus* 5 N isolate (OD_600_ = 0.5) after samples were treated with sublethal doses of aPDI (reduction in bacterial cell count ∼0.5 log_10_ units) using a Syngen Blood/Cell RNA Mini Kit (Syngen, Poland). The TranScriba kit (A&A Biotechnology, Poland) was used to transcribe the RNA to complementary DNA (cDNA). qPCR assays were performed using a LightCycler 480 II (Roche Life Science, Germany). The reaction mixture (10 μL total) consisted of 5 μL of Fast SG qPCR Master Mix (EURx, Poland), 200–400 nM of each primer (TIB MOLBIOL, Germany) (Table S1), nuclease-free water, and 1 μL of fivefold dilution of cDNA. The following steps were implemented (Table S2). Melting curve analysis was carried out to exclude primer-dimer formation or nonspecific amplification. Relative changes in the expression of the *sec*, *tst*, *srrA*, and *srrB* genes were normalized to the *gmk* reference gene.

### 2.12 Western Blot Immunodetection

*S. aureus* 5 N was grown in TSB until the logarithmic phase of growth was reached (OD_600_ = 1.5), diluted 10× in TSB, and then treated with sublethal conditions of aPDI using each of the test compounds to obtain a reduction in bacterial cell count of ∼1 log_10_ unit. After irradiation, bacterial supernatants were harvested 1 h after treatment. aPDI-treated and untreated supernatants were mixed 1:1 (v/v) with 2× Laemmli buffer (Bio-Rad, USA) supplemented with β-mercaptoethanol (Sigma-Aldrich, USA). Samples were heated to 95 °C for 5 min, centrifuged (13,200 rpm/min), and stored at −20 °C. The total protein concentration in the tested samples was determined with the RC DC Protein Assay kit I (Bio-Rad, USA) based on a standard curve prepared from the γ-globulin protein standard (Bio-Rad, USA). SEC or TSST-1 (Toxin Technology, Inc., USA) standard proteins and lysates were separated by SDS-PAGE at 180 V for 1 h and then wet transferred to PVDF membranes (Bio-Rad, USA) for 1 h at 100 V. The membrane was washed twice with TBS buffer (0.01 M Tris–HCl, pH 7.5, 0.05 M NaCl) and then incubated in TBS-T (TBS buffer with 0.5% (v/v) Tween 20, CHEMPUR, Poland) suspended in a 1% solution of skim milk powder for 30 min. After washing with TBS, the membrane was incubated with primary rabbit antibodies against the toxins (1:10,000) (Toxin Technology, Inc., USA) overnight at 4 °C with gentle shaking. The membrane was then washed three times with TBS and incubated with anti-rabbit alpaca secondary antibodies labeled with HRP (1:10,000 in TBS-T with 1% milk) (Jackson ImmunoResearch Laboratories Inc., USA) for 30 min with shaking at room temperature. Excess antibodies were removed by washing three times with TBS-T for 5 min, and residual detergent was removed by washing twice with TBS. Membranes were placed in the ChemiDoc XRS+ gel documentation system and visualized using the Clarity Max membrane reagent (Bio-Rad, USA).

### 2.13 IL-2 ELISA

Human PBMCs were counted, diluted in RPMI-1640 medium, and seeded at 1 × 10^5^ per well in a 96-well round-bottom plate (Corning, USA). aPDI-treated or untreated SEC or TSST-1 toxin (80 ng/mL) was added to PBMCs and incubated for 24 h at 37 °C in a 5% CO_2_ atmosphere. Afterward, the plate was centrifuged (300 × *g*/5 min/4 °C), and supernatants were collected and kept at −80 °C for further analysis. IL-2 production measurements in each condition were determined with an IL-2 Human Uncoated ELISA Kit (Invitrogen, USA) according to the manufacturer’s protocol. Three independent biological replicates of PBMCs derived from three different donors with three technical repetitions of aPDI were used in this experiment. The absorbance at 450 nm was measured, and the signal was calculated as IL-2 production based on the standard curve of human IL-2. As a positive control, PBMCs were chemically treated with 150 ng/mL phorbol 12-myristate 13-acetate (PMA) (Sigma-Aldrich, USA) and 75 ng/mL ionomycin (Sigma-Aldrich, USA).

### 2.14 Statistical Analysis

Statistical analysis was performed using GraphPad Prism 9 (GraphPad Software, Inc., CA, USA). Quantitative variables were characterized by the arithmetic mean and the standard deviation of the mean. Data were analyzed using either one-way or two-way ANOVA with Dunnett’s multiple comparison test.

## 3 Results

### 3.1 Ga^3+^CHP Is an Efficient Photogenerator of ROS under Green Light Illumination

**Figure 3:**
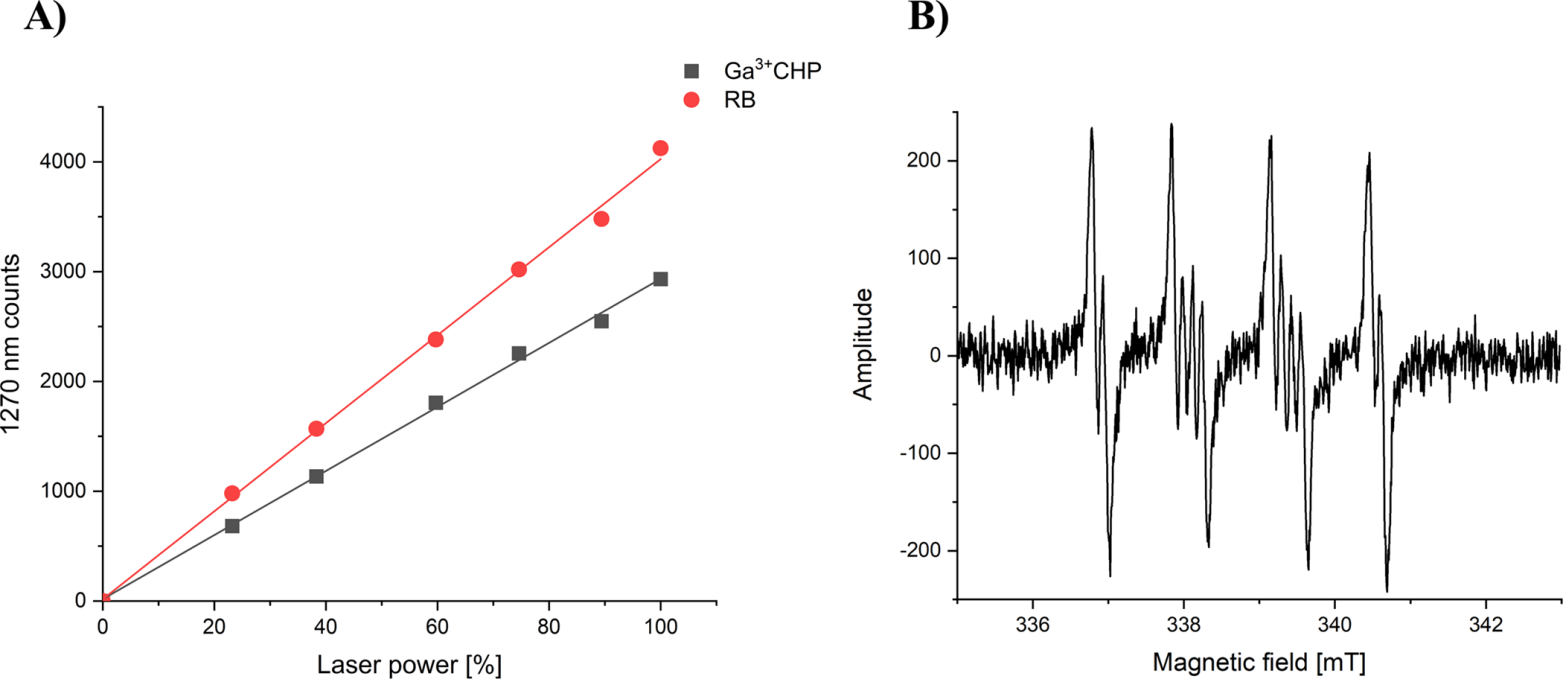
Photodynamic properties of Ga^3+^CHP under green light illumination. (A) Efficiency of singlet oxygen photogeneration by Ga^3+^CHP compared to that by the standard RB. Measurements of singlet oxygen photogeneration were performed in deuterium oxide (D_2_O). Samples were excited at 540 nm laser light. Maximum power density was ca. 7 mW/cm^2^; the laser emits the beam at a frequency of 1 kHz, which gives about 7 μJ/cm^2^ of each pulse (this is as 100% on the graph). (B) Detection of the superoxide anion generated by Ga^3+^CHP by EPR spin trapping using DMPO as a spin trap dissolved in 75% DMSO.

We recently synthesized Ga^3+^CHP, a novel antimicrobial compound, which comprises a porphyrin ring moiety, a Ga^3+^ metal ion, and two quaternary ammonium groups at the ends, which significantly increased the solubility of the compound in water compared to that of protoporphyrin IX loaded with gallium ions (Ga^3+^PP) (40.3 mg mL^–1^ for Ga^3+^CHP vs <0.1 mg mL^–1^ for Ga^3+^PP) (Figure 3).^21^ Here, we were interested in whether the newly synthesized Ga^3+^CHP efficiently produces ROS upon visible light excitation (522 nm). The quantum yield of singlet oxygen photogeneration of Ga^3+^CHP in comparison to the singlet oxygen photogeneration of standard RB is shown in Figure 3A. Ga^3+^CHP exhibited singlet oxygen photogeneration at a yield of 0.55, indicating that during photodynamic action, this compound efficiently generates singlet oxygen. Furthermore, by EPR spin trapping, we found a spin adduct with spectral parameters consistent with that of DMPO-OOH, indicating the photogeneration of superoxide anions, although at very low yield, after green light irradiation with Ga^3+^CHP in a mixture of 75% DMSO (Figure 3B). The results suggested that Ga^3+^CHP is mainly a type II photosensitizer that uses the energy transfer from the triplet state of the PS to produce highly toxic singlet oxygen (^1^O_2_). Ga^3+^CHP also generated superoxide anions at a low but detectable level.

### 3.2 Ga^3+^CHP Is Recognized and Accumulated by Heme-Specific Receptors and a Heme-Specific Efflux Pump

**Figure 4:**
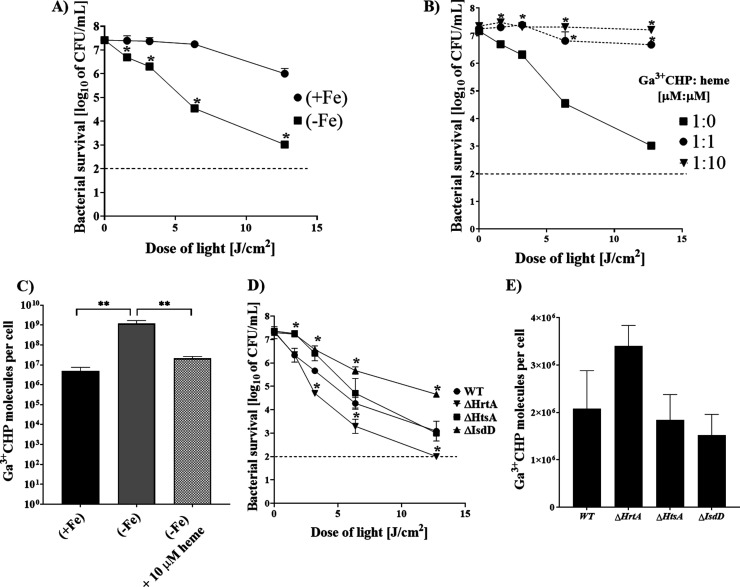
Dependence of the Ga^3+^CHP-mediated aPDI effect and intracellular accumulation on heme and iron. Experiments were conducted using two *S. aureus* strains: 25923 (A–C) and Newman (WT) together with its isogenic mutants (ΔHrtA, ΔIsdD, and ΔHtsA) (D, E) in the presence (+Fe) or absence (−Fe) of iron. (A) Effect of aPDI on bacterial survival after treatment with 1 μM Ga^3+^CHP and green light in the presence or absence of iron. Significance at the respective p values is indicated with asterisks (*p < 0.001). *S. aureus* 25923 cells cultured in the presence of iron (+Fe) were used as a control. (B) Effect of aPDI on the different ratios of Ga^3+^CHP to the natural ligand heme [μM:μM]. Prior to the experiment, bacterial cells were cultivated in medium without iron. (C) Intracellular accumulation of 10 μM Ga^3+^CHP in *S. aureus* cultured with varying iron levels in the medium and after the addition of 10 μM heme (corresponding to a PS:heme concentration ratio of 1:1). Significance at the respective *p* values is indicated with asterisks (**p < 0.005). *S. aureus* 25923 cells cultured in the absence of iron (−Fe) were used as a control. (D) Phototreatment of the wild-type strain or deletion mutants (ΔHrtA, ΔIsdD, and ΔHtsA) with 1 μM Ga^3+^CHP under green light irradiation (522 nm) in the presence of iron in the medium. Significance at the respective p values is indicated with asterisks (*p < 0.001) for *S. aureus* Newman WT. (E) Ga^3+^CHP uptake at 1 μM by the Newman *S. aureus* strain and its isogenic mutants in the presence of iron in the medium. All experiments were conducted in three biological replicates, and the data are presented as the mean ± SD. The dashed line (A, B, D) at 2 log_10_ CFU/mL is the detection limit of the test.

The modification of the core porphyrin ring with two positively charged quaternary ammonium groups equipped the molecule with the ability to efficiently bind negatively charged bacterial surface, allowing the cells to efficiently accumulate the PS via electrostatic interactions. We further wanted to investigate whether Ga^3+^CHP could be actively accumulated by staphylococcal cells similar to its structural analogue—heme. We tested this in several experiments: (i) analysis of aPDI in conditions of iron availability or absence, (ii) intracellular accumulation of compounds, and (iii) use of mutants with disabled heme transport proteins. We examined the effect of aPDI with 1 μM Ga^3+^CHP and green light illumination on bacterial survival using divergent iron availability in the environment (Figure 4A). Iron starvation potentiated the aPDI effect, showing a reduction in bacterial survival of 4.5 log_10_ CFU/mL compared to bacteria cultured in the presence of iron, showing a reduction in survival of only 1.5 log_10_ CFU/mL. Literature data indicated that in the absence of iron, elevated production of heme transport proteins by bacterial cells was observed; as a consequence, more of the compound accumulated in the cells,^28^ which in our case resulted in increased aPDI efficiency. Higher aPDI efficiency in iron-starved bacteria was reversed by the addition of iron-containing heme. The addition of the same concentration (1 μM) or a 10-fold excess of heme significantly reduced the effect of Ga^3+^CHP-mediated aPDI, indicating that both compounds (heme and Ga^3+^CHP) compete for the same heme transport proteins (Figure 4B).

This was confirmed by the results of Ga^3+^CHP accumulation, which was significantly reduced in bacteria in the presence of iron compared to lack of iron. Addition of heme, competing with Ga^3+^CHP for binding to heme transport proteins, resulted in a significant reduction of Ga^3+^CHP accumulation in an iron-depleted medium, confirming that Ga^3+^CHP is recognized by heme transport proteins (Figure 4C).

We further investigated the role of selected heme acquisition (Isd or Hts) and heme detoxification (HrtAB efflux pump) systems in aPDI (Figure 4D). Staphylococcal cells with an impaired heme efflux pump (ΔHrtA) showed the most sensitive phenotype to Ga^3+^CHP-mediated aPDI, presenting a decrease in bacterial survival by 5.3 log_10_ CFU/mL (Figure 4D). ΔIsdD cells lacking a functional heme acquisition mechanism were the most tolerant to aPDI treatment among the studied mutants, with only a 2.6 log_10_ CFU/mL reduction in bacterial counts. This finding was also reflected in intracellular accumulation of Ga^3+^CHP, where efflux pump impairment showed the greatest accumulation of the compound, while the cells without IsdD demonstrated the lowest accumulation (Figure 4E). In this case, we observed a difference in accumulation, but statistical significance was not reached. These results showed that heme-specific Isd receptors and the HrtAB efflux pump may be important for Ga^3+^CHP accumulation and phototreatment.

### 3.3 Ga^3+^CHP Effectively Photosensitizes *S. aureus* Isolates during Phototreatment

**Figure 5:**
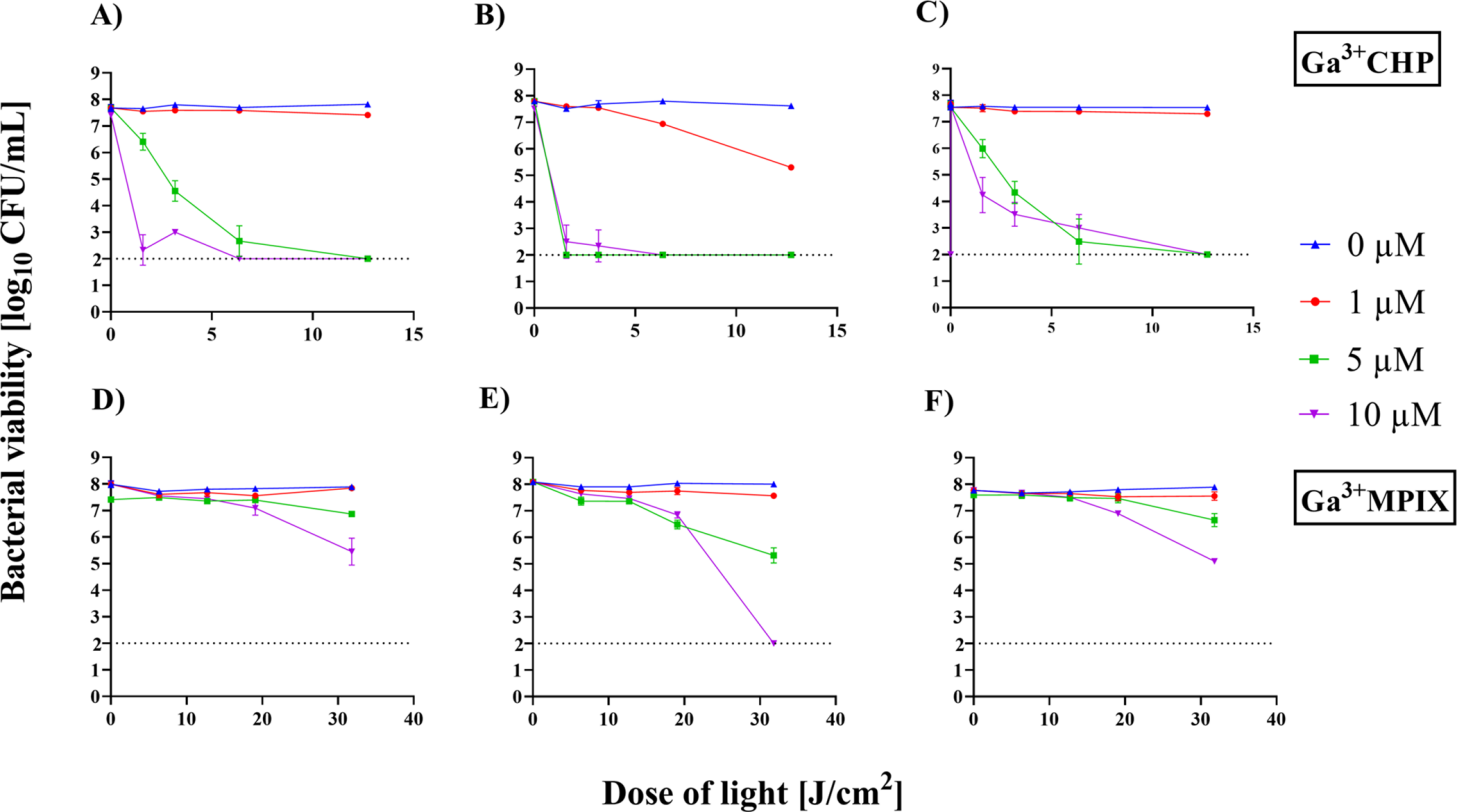
Photoinactivation of *S. aureus* isolates from patients with atopic dermatitis. Two photosensitizers, Ga^3+^CHP (A–C) and Ga^3+^MPIX (D–F), activated with green (522 nm) light were used to evaluate the effectiveness of aPDI on the survival of 3 N (A, D), 5 N (B, E) and 38 N (C, F) isolates. The detection limit was 100 CFU/mL (dashed lines). Each experiment was performed in three independent biological replicates. The data are presented as the mean ± SD of three separate experiments. The dashed line at 2 log_10_ CFU/mL is the detection limit of the test.

To determine whether photoinactivation with Ga^3+^CHP is an effective treatment for reducing *S. aureus* colonization in patients with AD, we first investigated its efficacy against three clinical isolates of *S. aureus* (Figure 5). Photoinactivation of bacterial cells with 5 μM Ga^3+^CHP followed by irradiation (12.7 J/cm^2^) resulted in a reduction in the number of bacteria by ∼5.5 log_10_ CFU/mL for all tested strains. The concentration of the reference metalloporphyrin Ga^3+^MPIX had to be increased to 10 μM and the light dose to 31.8 J/cm^2^ to observe a decrease in the number of bacterial cells of the 5 N isolate below the detection limit, while for the other two isolates tested, 3 and 38 N reached a lethal efect (for 3 N – 2.5 log_10_ CFU/mL; for 38 N – 2.6 log_10_ CFU/mL). The PS concentrations and doses of green light used to achieve effective aPDI significantly differed between the two compounds. A sufficient reduction in bacterial cell number was obtained by Ga^3+^CHP-mediated aPDI using suitable conditions, and the irradiation time was shorter for Ga^3+^CHP-mediated aPDI than for Ga^3+^MPIX-mediated aPDI. A strain-dependent response to aPDI was also observed, with the 5 N isolate being the most sensitive to aPDI regardless of the PS used. We also tested the effectiveness of both compounds against the biofilm formed by the clinical isolate of *S. aureus* 5 N. In this case, we observed a Ga^3+^CHP concentration- and light dose-dependent reduction in the number of biofilm-forming cells. In the case of biofilms, aPDI using Ga^3+^CHP proved significantly more effective than Ga^3+^MPIX (Figure S3).

**Figure 6:**
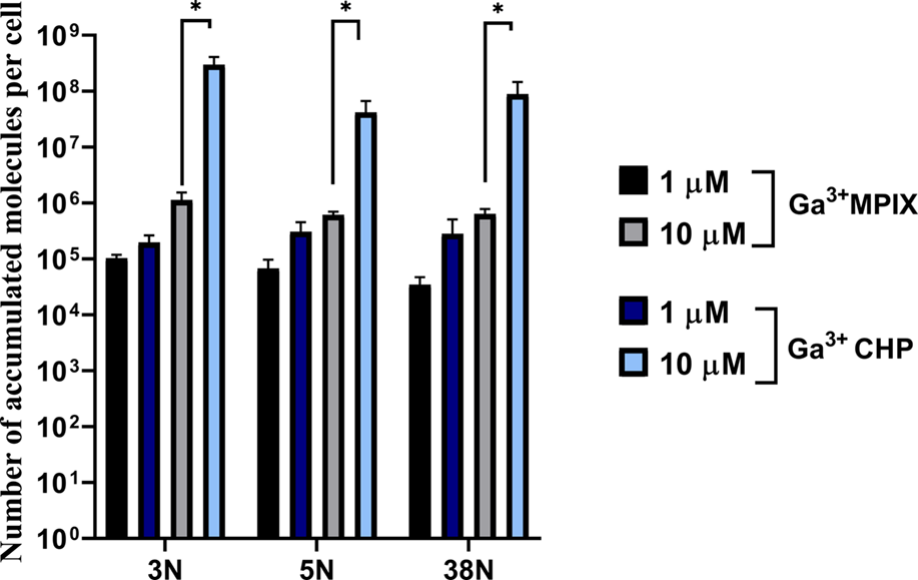
*S. aureus* accumulation of the photosensitizers. The *S. aureus* strains 3, 5, and 38 N were exposed to either Ga^3+^MPIX or Ga^3+^CHP for 10 min with shaking at 37 °C. Then, the cells were washed twice to eliminate extracellular PS and resuspended in lysis buffer. After 24 h of incubation in the dark, the fluorescence of the lysates was measured on a plate reader. The data are presented as the mean of accumulated PS molecules from three biological repetitions, calculated based on the standard curve of each compound in the lysis buffer.

To explain the superior efficacy of Ga^3+^CHP-mediated aPDI compared to that of Ga^3+^MPIX-mediated aPDI, we compared the intracellular accumulation of tested compounds across all studied strains (Figure 6). Incubation of the cells with 1 μM PSs resulted in the accumulation of 10^4^–10^5^ and 10^5^ molecules per cell for Ga^3+^MPIX and Ga^3+^CHP, respectively. After increasing the concentrations of the tested compounds to 10 μM, Ga^3+^CHP was strongly accumulated in each tested strain, reaching an order of magnitude of 10^7^–10^8^ molecules per cell. In comparison, the accumulation of Ga^3+^MPIX at the same 10 μM concentration remained in the range of 10^5^–10^6^ molecules per cell. We also evaluated the accumulation of both compounds in the *S. aureus* strain 25923 using fluorescence confocal microscopy (Figure S2). Compared to Ga^3+^MPIX, Ga^3+^CHP accumulated at significantly higher levels, which was reflected by its higher fluorescence intensity. Undoubtedly, the Ga^3+^CHP accumulation was higher than that of Ga^3+^MPIX in all tested strains, which explains its higher efficiency in aPDI.

### 3.4 Ga^3+^CHP-Based Photosensitization Reduced the Viability of *S. aureus* in an Ex Vivo Porcine Skin Model

**Figure 7:**
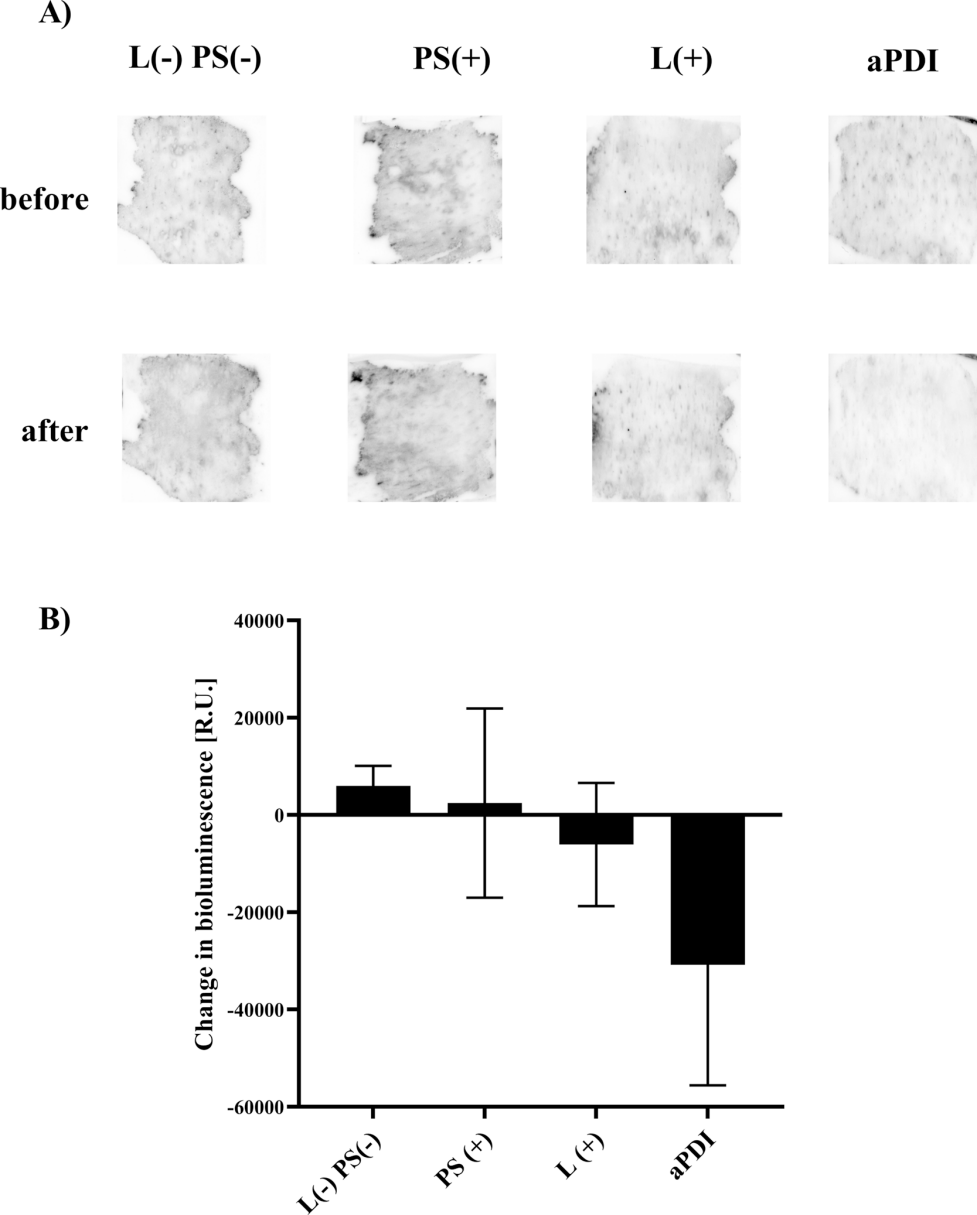
Evaluation of Ga^3+^CHP-mediated aPDI treatment against the *S. aureus* strain Xen40 in an ex vivo porcine skin model. (A) Bioluminescent *S. aureus* strain was applied to clean porcine skin grafts 24 h before treatment. Ga^3+^CHP was applied for 20 min and incubated at 37 °C before irradiation (12.72 J/cm^2^). The bioluminescence signal was measured before and immediately after aPDI treatment (10 μM Ga^3+^CHP, 12.72 J/cm^2^). (B) Bioluminescence was measured by ChemiDoc and Bio-Rad instruments and calculated by ImageJ software. The change in the bioluminescent signal was measured for each condition, and the average of three independent biological replicates is presented in the graph. The data are presented as the mean ± SD of before vs after treatment.

To evaluate whether the significant reduction in viability obtained in vitro could be translated into a more complex biological system, we applied an ex vivo porcine skin model. In this experiment, we used the bioluminescent *S. aureus* strain Xen40 for the colonization of porcine skin ex vivo. After establishing a biofilm on the surface of the skin (24 h post bacteria application), Ga^3+^CHP was added and aPDI was performed. The viability of bacteria was determined by measuring the bioluminescence signal of *S. aureus* before and after treatment with Ga^3+^CHP alone, light alone, or combined treatment (Figure 7A). The change in the bioluminescence signal was calculated and is presented in Figure 7B. Untreated cells and cells treated with Ga^3+^CHP showed only slightly altered bioluminescence signals, indicating that the bacteria were still present on the skin. In contrast, the bioluminescence signal decreased after aPDI treatment, indicating a reduction in the viability of bacterial cells. Light-only treatment also exhibited a decrease in the bioluminescence signal, but the decrease was not as severe as that resulting from aPDI treatment. Ga^3+^CHP combined with green light irradiation might be a promising method for the reduction of *S. aureus* colonization on the skin. Although measurement of the bioluminescent signal indicated differences between aPDI treated and untreated samples, the values of the measured signal did not reach statistical significance. On the other hand, the direct method of counting bacterial cells before and after aPDI treatment indicated a statistically significant difference between the number of cells after aPDI treatment compared to cells treated only with Ga^3+^CHP or treated only with light (Figure S4).

### 3.5 Ga^3+^MPs Are Not Mutagenic under Light or Dark Conditions

**Figure 8:**
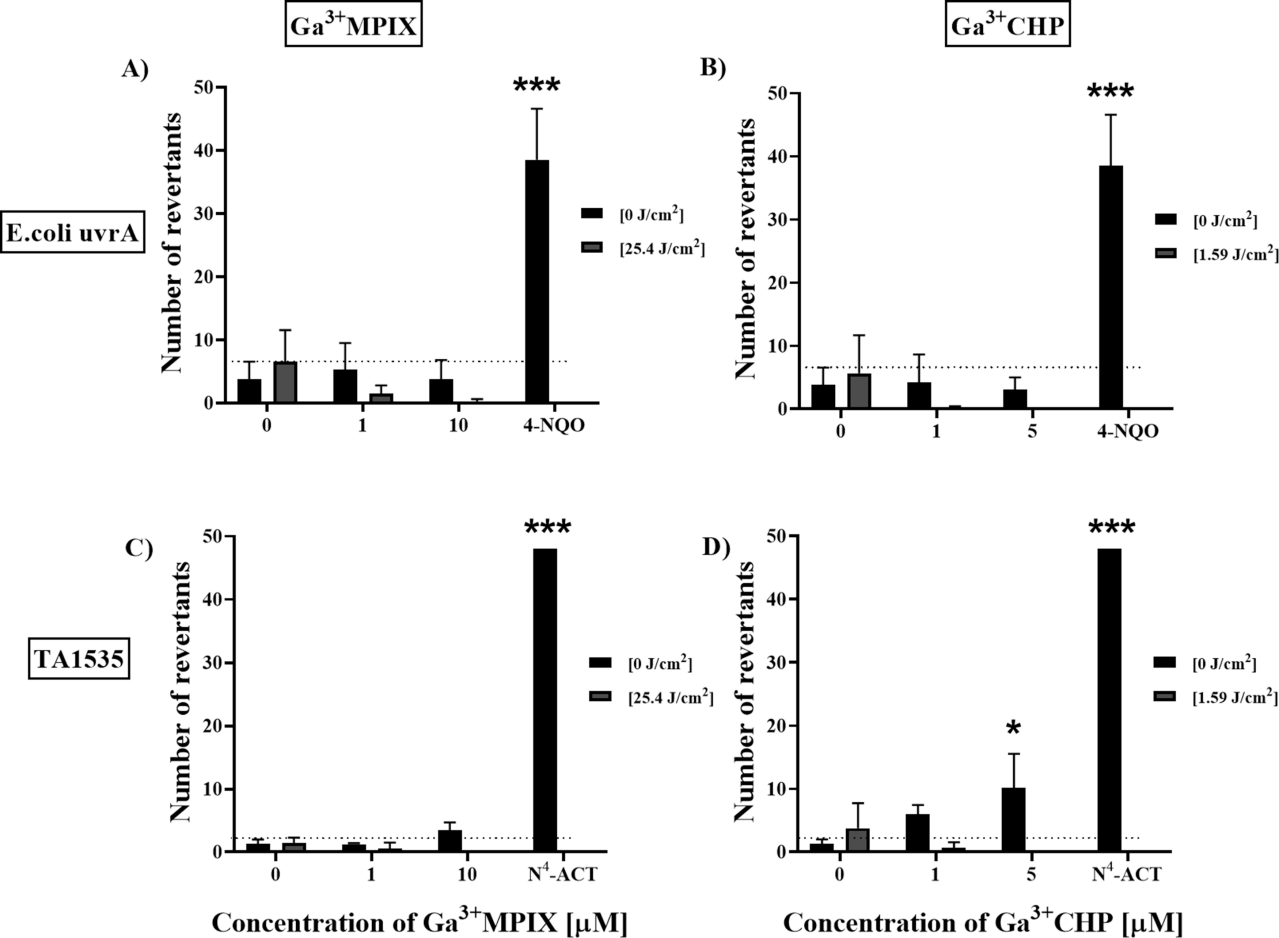
Examination of mutagenicity of photoinactivation with gallium metalloporphyrins. *E. coli* uvrA (A, B) and TA1535 (C, D) strains were exposed to both gallium compounds: Ga^3+^MPIX (A, C) or Ga^3+^CHP (B, D) either in the dark or under green light conditions. Additionally, two types of controls were included: untreated cells (0 μM, 0 J/cm^2^) as the negative control and chemically induced revertants with 90 min incubation with mutagens 4-NQO for *E. coliuvrA* or N^4^-ACT for TA1535 as positive controls. All treatment groups were incubated with mutagen or gallium compound for 90 min at 37 °C. For the light-activated treatment groups, after 10 min of incubation with compounds, cells were exposed to green light at the proper dosage and then incubated for 90 min. Then, exposure medium was added to the incubated cultures, and the samples were divided into 384-well plates with each sample being distributed to 48 wells with three technical repetitions. The microplates were incubated for 48 h at 37 °C. The assessment of revertants was conducted by determining the change in exposure medium color (from blue to yellow) in each well. Yellow color represented the occurrence of revertants. The experiment was performed in three biological replicates with three technical replicates of each treatment group in each experiment. Data are presented as the mean ± SD of the number of revertants. The dashed line indicates the level of spontaneously formed revertants (baseline). Cyto- and phototoxicity of aPDI against indicator strains are presented in the Supporting Information (Figure S5).

To assess the safety of aPDI treatment with Ga^3+^MPs, we tested the mutagenic potential of both gallium compounds on two reference bacterial strains designed to quantify the mutagenic potential of various compounds, namely, *Escherichia coli* uvrA and *Salmonella typhimurium* TA1535. We tested both compounds, Ga^3+^MPIX and Ga^3+^CHP, with or without exposure to green light to test the potential mutagenicity of the compounds independent of light and in a light-dependent manner (aPDI treatment). We applied two types of aPDI conditions: (i) mild, in which the reduction in *S. aureus* cell number did not exceed 1 log_10_ CFU/mL, and (ii) strong, in which the reduction in *S. aureus* cell number was at least 2 log_10_ CFU/mL. Thus, for each compound, the aPDI conditions were different. At the same time, the aPDI doses would have to be sublethal for the indicator strains (*E. coli* and *S. typhimurium*) in order for us to determine the number of revertants formed (Figure S5). Based on the *E. coli* uvrA strain analysis data, we did not observe an increased number of revertants when using either compound under light activation or dark conditions (Figure 8A,B). In the case of *S. typhimurium* indicator strain TA1535, we observed an increased number of revertants at 5 μM concentration: for Ga^3+^CHP in the dark conditions (Figure 8C,D). It is noteworthy that the number of revertants obtained after treatment slightly exceeded the baseline; however, these results are still significantly lower than for the chemically induced positive control (Figure 8C,D). After excitation of Ga^3+^CHP with light, the number of revertants never exceeded the baseline. The results obtained indicated that both compounds were not mutagenic after light activation, while the observed increased number of revertants after treatment in the dark would require more in-depth analyses to conclusively resolve the safety of Ga^3+^CHP.

### 3.6 Ga^3+^CHP Is less Phototoxic Than Ga^3+^MPIX to Human Keratinocytes and Is Not Dependent on Filaggrin Levels

**Figure 9:**
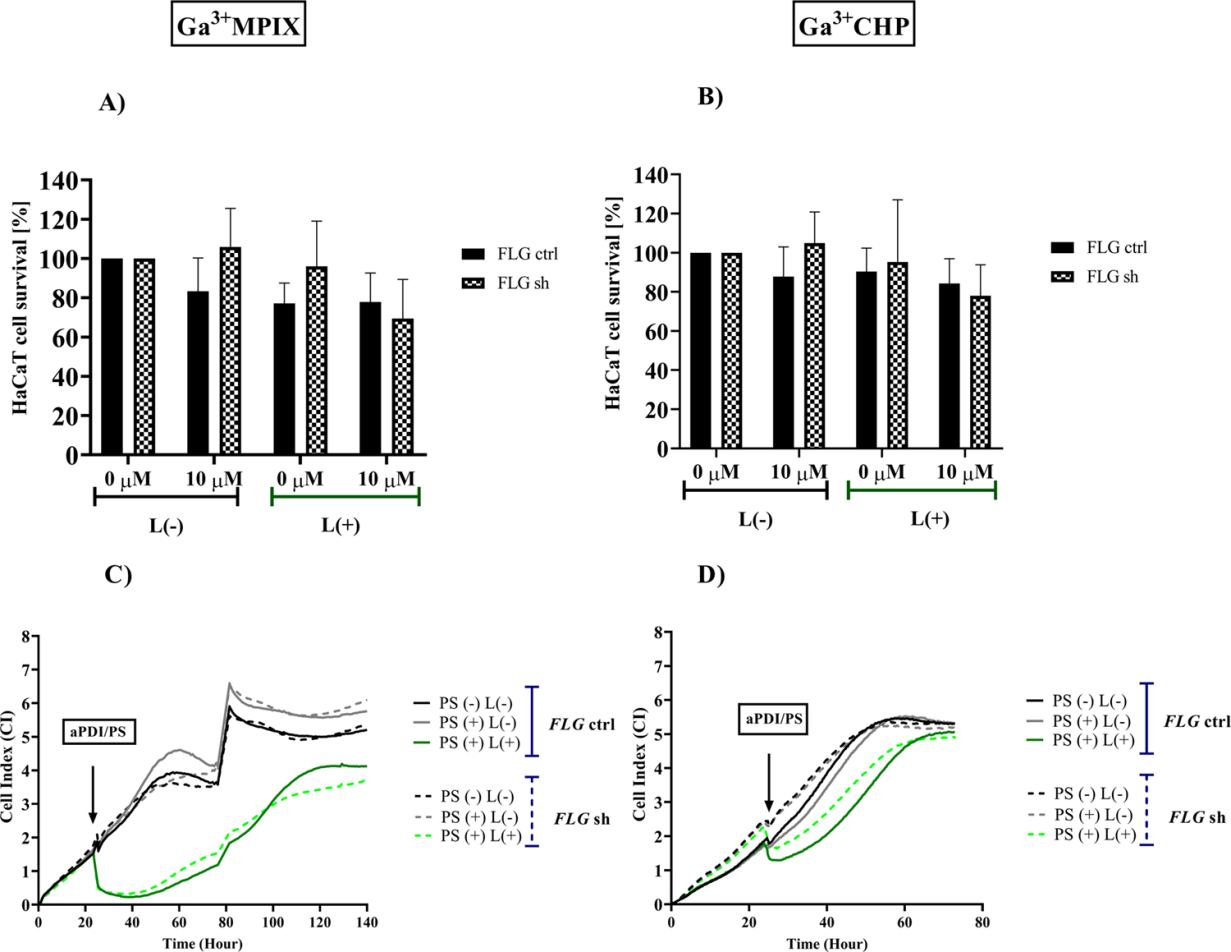
Effect of photoinactivation with Ga^3+^MPIX and Ga^3+^CHP on human keratinocytes with divergent filaggrin (FLG) expression. (A, B) Viability of HaCaT cells with normal filaggrin expression (*FLG* ctrl) and filaggrin knockdown (*FLG* sh) was measured with the MTT assay after dark or light exposure to Ga^3+^MPIX (31.9 J/cm^2^) (A) or Ga^3+^CHP (1.59 J/cm^2^) (B). (C, D) Cell growth dynamics of both lines: *FLG* control (*FLG* ctrl) or cells impaired in filaggrin expression—(*FLG* sh) after dark/light exposure to 10 μM Ga^3+^MPIX (31.9 J/cm^2^) (C) or Ga^3+^CHP (1.59 J/cm^2^) (D). The CI (represented as the *Y*-axis) was measured for each condition every 10 min. The *x*-axis shows the experimental duration in hours. The values presented are the average of the 7 technical repetitions. PS (+/−) refers to the presence/absence of a photosensitizer and L (+/−) to light.

To determine whether the aPDI conditions used for efficient photoinactivation of bacterial cells are toxic to eukaryotic cells, we tested them on HaCaT human keratinocytes (i) with normal filaggrin expression (*FLG* ctrl) and (ii) with filaggrin suppression (*FLG* sh). The HaCaT *FLG* sh cell line was used as a model of atopic skin as the expression level of filaggrin in AD patients is significantly reduced.^11^ According to the MTT assay results, the cytotoxicity of HaCaT cells with both normal and aberrant *FLG* expression was negligible as the viability of both cell groups was in the range of 83–105% compared to that of untreated cells. Regarding phototoxicity, the viability of both cell lines was estimated to be ≥78% 24 h after aPDI treatment according to the MTT assay, which is considered an acceptable toxicity value (Figure 9A,B). Since the MTT assay only measures toxicity at a selected measurement point, we used the xCELLigence technique to investigate the cell growth and proliferation dynamics of both cell lines after aPDI treatment. Ga^3+^MPIX-mediated aPDI inhibited the proliferation and growth of both cell lines (*FLG* ctrl and *FLG* sh), and the number of surviving cells reached a plateau after approx. 140 h of the experiment, however, without reaching the cell index (CI) value of untreated cells (Figure 9C). In comparison, the Ga^3+^CHP-mediated aPDI group showed significantly lower phototoxicity, with both cell groups reaching a plateau and a CI value equal to untreated cells after only 70 h, which was twice as fast as Ga^3+^MPIX-mediated aPDI (Figure 9D). Interestingly, compared to *FLG* ctrl HaCaT cells, *FLG* sh HaCaT cells showed slightly faster growth after aPDI treatment. Both gallium compounds excited with green light reduced the viability and proliferation rate of human keratinocytes; however, only cells treated with Ga^3+^CHP were viable enough to reach the plateau phase in a time period similar to that of untreated cells. It is worth mentioning that we did not observe cytotoxicity when the compounds were tested in dark conditions.

### 3.7 Effect of aPDI with Ga^3+^MPs on SEC Superantigen Expression, Production, and Biological Functionality

**Figure 10:**
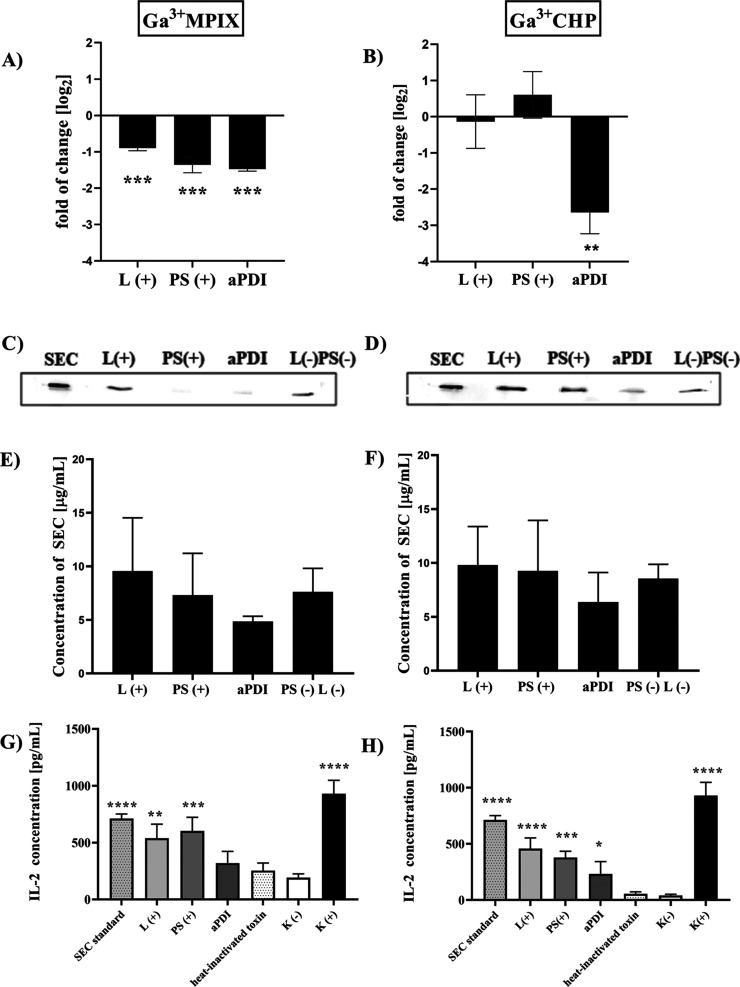
Effect of photoinactivation with gallium metalloporphyrins on gene expression, protein production, and biological activity of SEC. (A, B) Relative gene expression of *sec* normalized to the reference gene *gmk*. Cells were diluted 1:100 and grown until the OD_600_ was 0.5, incubated with the proper photosensitizer–10 μM Ga^3+^MPIX (A) or 1 μM Ga^3+^CHP (B) for 10 min and illuminated with 522 nm light (12.7 J/cm^2^ for Ga^3+^MPIX or 1.52 J/cm^2^ for Ga^3+^CHP). The expression of *sec* was measured in three biological samples with three technical repetitions of each sample. Error bars represent the standard error of the mean (SEM) values. (C–F) Western blot analysis after each treatment in the presence or absence of 522 nm light and Ga^3+^MPIX (C) or Ga^3+^CHP (D). Supernatants were harvested 1 h after aPDI treatment, and 10 μg of supernatant, calculated by the modified Lowry protocol, was added to the gel for each treatment. The intensity of the band was measured by ImageJ software and calculated according to the SEC protein standard curve. (G, H) IL-2 measurements from activated PBMCs exposed to SEC toxin untreated or pretreated with aPDI or 10 μM Ga^3+^MPIX with 12.72 J/cm^2^ (G) or 2 μM Ga^3+^CHP with 6.36 J/cm^2^ (H). The toxin was also exposed to light alone (L+) or photosensitizer alone (PS+), where the controls were heat-inactivated toxin (incubated for 1 h at 99 °C), K(−)—PBMC cells not exposed to toxin and K(+)—chemically activated PBMC cells. Significance at the respective p values is marked with asterisks [*p < 0.05; **p < 0.01; ***p < 0.001, ****p < 0.0001 with respect to untreated samples (cells maintained in dark conditions)].

*S. aureus* has been shown to colonize the skin surface of AD patients, promoting its pathogenicity by producing a number of virulence factors, such as SEC or TSST-1 superantigens.^29,30^ We investigated whether Ga^3+^MPs in combination with light could affect the expression level, production, or biological functionality of these superantigens. A decrease in *sec* expression levels was observed not only after aPDI treatment (a decrease of 1.47 log_2_ units) but also after treatment with light alone (a decrease of 0.89 log_2_ units) and photosensitizer alone (1.35 log_2_ units) compared to untreated cells (Figure 10A). In contrast, after Ga^3+^CHP-mediated aPDI, a significant downregulation of *sec* expression was observed (2.6 log_2_ unit decrease), whereas light alone or compound alone did not alter *sec* expression (Figure 10B). At the protein level, both light-activated Ga^3+^MPs only slightly reduced SEC production (no statistical significance) (Figure 10C–F), probably due to the insufficient sensitivity of the Western blot technique used in these analyses. However, we did not observe any significant difference between treatment with Ga^3+^MPIX (Figure 10C,E) or with Ga^3+^CHP (Figure 10D,F). Next, we were interested to know whether light excitation of Ga^3+^MPs affected the biological activity of SEC. In this experiment, SEC toxin was subjected to aPDI with Ga^3+^MPIX (Figure 10G) or Ga^3+^CHP (Figure 10H), and then SEC activity was evaluated in human peripheral mononuclear cells (PBMCs) after exposure to treated SEC. Bacterial superantigens, such as SEC and TSST-1, stimulate strong nonspecific activation and proliferation of lymphocytes resulting in the production of a large amount of cytokines.^31^ We tested superantigen activity by measuring interleukin 2 (IL-2), which is produced as a proinflammatory factor in response to SEC and TSST-1.^32^ The biological activity of SEC, as measured by IL-2 levels, was significantly reduced after treatment with Ga^3+^MPIX-mediated aPDI (319 pg/mL) compared to untreated toxin (712.5 pg/mL). The level of IL-2 after PBMC exposure to the aPDI-treated toxin decreased to the level of IL-2 produced by cells treated with heat-inactivated SEC or cells not exposed to the toxin. Moreover, treatment with light or a photosensitizer alone had no effect on the proinflammatory activity of SEC. Similarly, IL-2 levels were also reduced by aPDI with Ga^3+^CHP-treated toxin (Figure 10H) compared to the untreated enterotoxin-, L(+)-alone, or PS(+)-alone treated toxin. Photoinactivation with both Ga^3+^MPs reduced the biological functionality of the SEC superantigen, although it did not significantly alter the total protein levels. Moreover, only Ga^3+^CHP-mediated aPDI significantly affected *sec* gene expression levels.

### 3.8 Phototherapy with Both Ga^3+^MPs Affects the Biological Function of TSST-1

**Figure 11:**
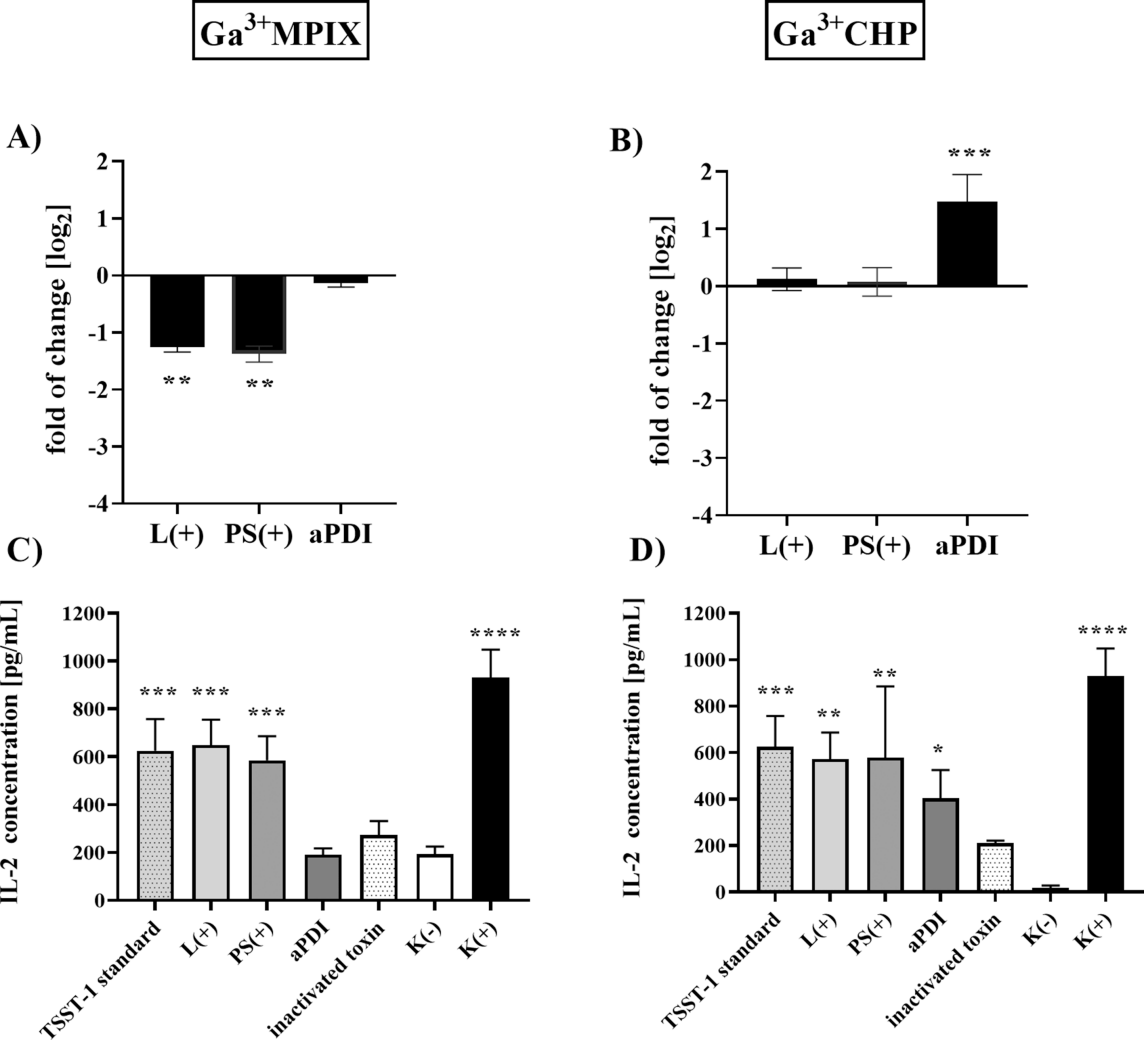
Effect of photoinactivation with gallium metalloporphyrins on gene expression, protein production, and biological activity of TSST-1. (A, B) Relative expression of the *tst* gene was normalized to the *gmk* reference gene and measured by quantitative PCR. Cells were diluted 1:100 and grown until the OD_600_ was 0.5, incubated with the proper photosensitizer—10 μM Ga^3+^MPIX (A) or 1 μM Ga^3+^CHP (B) for 10 min and illuminated with 522 nm light (12.7 J/cm^2^ for Ga^3+^MPIX or 1.59 J/cm^2^ for Ga^3+^CHP). The expression of *tst* was measured in three biological samples with three technical replicates each. Error bars represent SEM values. (C, D) IL-2 measurements from activated PBMCs exposed to TSST-1 toxin untreated or pretreated with aPDI: 10 μM Ga^3+^MPIX with 12.72 J/cm^2^ (G) or 10 μM Ga^3+^CHP with 12.72 J/cm^2^ (H). The toxin was also exposed to light alone (L+) or photosensitizer alone (PS+), where the controls were heat-inactivated toxin (incubated with 1 h 99 °C), K(−)—PBMC cells not exposed to toxin, and K(+)—chemically activated PBMC cells. Significance at the respective *p* values is marked with asterisks [*p < 0.05; **p < 0.01; ***p < 0.001 with respect to untreated samples (cells maintained in dark conditions)].

The second important superantigen we selected for our analyses was TSST-1, a virulence factor strongly associated with the exacerbation of inflammation in atopic skin. We investigated the effects of both green light-activated gallium compounds on the gene expression, protein levels, and biological function of the TSST-1 toxin. Ga^3+^MPIX-mediated aPDI did not alter the expression of the *tst* gene (Figure 11A), while treatment with light or photosensitizer alone decreased *tst* expression levels 1.259 log_2_ units and 0.38 log_2_ units, respectively. Interestingly, Ga^3+^CHP-mediated aPDI significantly upregulated *tst* expression by 1.5 log_2_ units (Figure 11B), while treatment with light alone or photosensitizing compound alone had no effect on *tst* expression levels. Next, we tested the biological functionality of TSST-1 after aPDI treatment using each of the tested compounds in vitro by measuring the level of IL-2 secreted by stimulated PBMCs, similar to the SEC study. TSST-1 treated with Ga^3+^MPIX-mediated aPDI reduced the level of IL-2 produced by activated PBMCs to levels estimated for nonactivated cells or after exposure to heat-inactivated TSST-1 toxin (Figure 11C). A reduction in IL-2 production in activated PBMCs was also observed after the TSST-1 toxin was treated with Ga^3+^CHP-mediated aPDI (Figure 11D). Pretreatment of TSST-1 with both compounds in the dark or light alone did not reduce IL-2 production, indicating that only aPDI has an effect on the superantigenic activity of TSST-1.

We did not quantify the level of the TSST-1 protein after aPDI treatment due to the low level of production of this protein. The in vitro biological functionality of the superantigen of TSST-1 was examined after both treatments.

### 3.9 Expression of the *tst* Gene Is Positively Correlated with the Expression of the srrAB Regulatory Genes after aPDI

**Figure 12:**
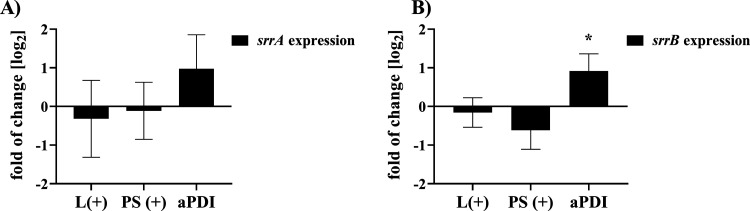
Effect of photoinactivation with gallium metalloporphyrin on the expression of the genes in the two-component SrrAB system. (A, B) Relative expression of the *srrA* (A) and *srrB* (B) genes normalized to the *gmk* reference gene. Cells were diluted 1:100, grown until the OD_600_ was 0.5, incubated with a photosensitizer (1 μM Ga^3+^CHP) for 10 min, and irradiated with light at 522 nm (1.59 J/cm^2^). The expression of both genes was measured in three biological repetitions with three technical replicates in each. The data are presented as the mean ± SEM.

The result of upregulated expression of the *tst* gene after Ga^3+^CHP aPDI was unexpected. To understand the mechanism of the observed upregulation, we investigated the genes of the SrrAB regulatory system, which modulates the production of the TSST-1 toxin in response to the presence of ROS in the environment.^33^ The expression of the *srrA* and *srrB* genes was investigated after Ga^3+^CHP phototreatment (Figure 12). We observed that the expression levels of both the *srrA* and *srrB* genes increased after aPDI treatment, which is consistent with the observed upregulation of the *tst* gene after photoinactivation. The expression of the TSST-1 toxin gene is positively correlated with the expression of the SrrAB regulatory system genes after aPDI.

## 4 Discussion

The skin microbiome differs between AD patients and healthy individuals. There is a significant increase in commensal bacterial load in the skin microbiome of AD patients, and *S. aureus* is a key pathogen in AD.^34^ Methods for staphylococcal decolonization in AD patients are mainly based on antibiotic therapies; however, due to increasing global antimicrobial resistance, antibiotic therapy remains a temporary solution. Most types of light-based treatments consist of ultraviolet A (UV-A) or narrow-band ultraviolet B (NB UV-B) exposure.^35^ UV-B treatment was shown to reduce the viability of colonized *S. aureus* on the skin of AD patients^36,37^ as 308 nm excimer light showed similar results.^38^ However, UV-B light penetrates through the epidermis layer of the skin, which generates a huge number of side effects and may promote carcinogenesis.^39^ Visible light-based therapies, such as antimicrobial photodynamic inactivation, are currently being considered to reduce *S. aureus* colonization on atopic skin.^2^ Photodynamic therapy (PDT) is currently approved in Europe and the USA for the treatment of actinic keratosis. The case study reported by Pozzi and Asero showed that PDT with a narrow-band red light (630 nm, 75 J/cm^2^) and 5-aminolevulinic acid (ALA) as a PS precursor can be successfully used in the treatment of AD patients. Three PDT sessions were used to treat skin lesions; however, the effect on *S. aureus* viability was not investigated.^40^ Red light penetrates deeper than UV light into the dermis layer; unfortunately, the pain effect can be a substantial obstacle in the case of AD patients with hypersensitive skin. Green light, on the other hand, penetrates the epidermis without causing as much pain, so the use of wavelengths in this spectral range may be a promising approach for the treatment of atopic skin.^41^ In this study, we have shown that Ga^3+^CHP can be effectively excited with green light, resulting in the production of singlet oxygen and to a lesser extent superoxide anion, which significantly reduced the viability of clinical *S. aureus* isolates derived from atopic skin (5 log_10_ CFU/mL for Ga^3+^CHP and 2.6 log_10_ CFU/mL for Ga^3+^MPIX). Additionally, we observed that aPDI treatment with Ga^3+^CHP effectively decolonized *S. aureus* in the ex vivo porcine skin model. Porcine skin has been used as the skin model due to its great similarity to human skin. This model has been applied in biofilm formation studies, skin barrier research, and wound infection models.^42,43^ However, studies on more complex models, such as ex vivo human atopic skin grafts or in vivo animal models, are needed.

Ga^3+^MPs are dual-function compounds that act according to light-independent and light-dependent mechanisms.^44^ In the light-independent mechanism, they are antimicrobial agents that slow bacterial growth by blocking iron metabolism based on their structural similarity to heme (Fe^3+^PPIX) in planktonic cultures and biofilms.^18,20,45,46^ These compounds can also act as photosensitizers in aPDI, as they are able to absorb visible light and, in the presence of molecular oxygen, photogenerate cytotoxic ROS.^17,20^ Porphyrins are a group of naturally occurring type II photosensitizers mainly producing singlet oxygen rather than radicals.^47^ The main disadvantage of porphyrin compounds is the formation of aggregates, resulting in poor solubility in aqueous solutions.^48,49^ The water solubility of antimicrobials has a great influence on their in vivo antimicrobial activity and biocompatibility in various tissues. Gallium porphyrin (Ga^3+^PP) is dissolved only in toxic organic solvents such as DMSO, whereas Ga^3+^MPIX is dissolved in an aqueous solution such as 0.1 M NaOH.^20^ The addition of two quaternary ammonium groups to the porphyrin core in the Ga^3+^CHP structure (Figure 1) significantly increased its solubility in water to 40.3 g mL^–1^, while the water solubility of Ga^3+^PP was <0.1 g mL^–1^.^21^ Despite the modification of the structure by the addition of ammonium groups, the Ga^3+^CHP structure remained similar to the heme structure, and it was still effectively accumulated by bacterial cells (Figures 6 and S2). Accumulation in bacterial cells is essential for the effective light-dependent and light-independent action of the compound. Bacterial cells contain heme acquisition receptors, the Isd system, and the HrtAB heme detoxification machinery, which seem to play a role in the sequestration and utilization of Ga^3+^CHP (Figure 4). Ga^3+^CHP-mediated aPDI efficacy, as well as its accumulation, strongly depended on heme or iron availability, further indicating that natural bacterial systems are responsible for Ga^3+^CHP uptake. ΔIsdD, a mutant in the transmembrane transporter, was the most tolerant to Ga^3+^CHP-mediated aPDI with the lowest Ga^3+^CHP accumulation, which indicated the predominant role of the Isd system in compound recognition and transmembrane transport. In our previous study on the meso-derivative Ga^3+^MPIX, we reported that Isd was involved in intracellular accumulation of Ga^3+^MPIX.^20^ It was also found that Isd receptors are involved in the uptake of Ga^3+^PPIX.^17^ Studies by Moriwaki et al. showed that there is stable binding between Ga^3+^MPs and IsdH receptors, which contain NEAT domains, supporting the hypothesis that the Isd system might play a role in intracellular accumulation.^50^ However, a thorough study of the receptor–ligand interaction for either Ga^3+^MPIX or Ga^3+^CHP needs to be conducted to evaluate whether such structural changes are crucial for compound recognition by the Isd system. To date, there is limited knowledge concerning the role of heme detoxification in the process of non-iron metalloporphyrin utilization. The expression of heme detoxification machinery genes was upregulated after exposure to Ga^3+^MPs, and the activation of this system is needed to partly overcome the toxicity of those compounds.^51^ We have previously shown that the ΔHrtA *S. aureus* mutant was more sensitive than the WT to aPDI treatment with various porphyrin compounds, e.g., PPIX or Ga^3+^MPIX.^20,26^ The current study revealed the same pattern for Ga^3+^CHP (Figure 4D). Without light exposure, Ga^3+^MPIX was revealed to be the most toxic for the ΔHrtA mutant among other compounds, such as Ga^3+^PPIX or PPIX.^20^ However, Wakeman et al. showed that there was no significant change in Ga^3+^PPIX accumulation between *S. aureus* WT and ΔHrtB despite higher cytotoxicity under dark conditions.^51^ In our case, ΔHrtA accumulated the highest level of Ga^3+^CHP molecules per cell among all tested phenotypes; however, there was no significant difference in comparison to the WT strain (Figure 4E). The same observation was made for Ga^3+^MPIX. All these findings support the hypothesis that HrtAB may not be a specific or the only export pump of Ga^3+^MP molecules; however, by blocking its activity, the toxicity of gallium compounds may increase in both light-activated and dark conditions. Since there are currently no HrtAB efflux pump blockers on the market, this field deserves to be explored due to its high application potential.

An ideal photosensitizer candidate should exhibit high phototoxicity with low toxicity in the dark, a high quantum yield of singlet oxygen production and/or other ROS photogeneration, and high safety in eukaryotic cells.^52^ In this study, we characterized the light-dependent mechanism of Ga^3+^CHP. In general, for porphyrins, the quantum yield of singlet oxygen generation is estimated to be between 0.5 and 0.8.^53^ Ga^3+^CHP is a type II photosensitizer that generates singlet oxygen with a quantum yield of 0.55 and produces a low level of the superoxide anion through electron transfer mechanisms (Figure 3). However, despite the lower quantum yield of singlet oxygen generation, Ga^3+^CHP was more efficient in aPDI than Ga^3+^MPIX (quantum yield of singlet oxygen generation—0.69) (Figure 5).^20^ The higher efficacy of Ga^3+^CHP-mediated aPDI in the reduction of *S. aureus* might be explained by the ∼10× higher intracellular accumulation of Ga^3+^CHP compared to that of Ga^3+^MPIX (Figure 6). PS localization near sensitive targets is a crucial factor for photodynamic efficacy due to the short diffusion length of photogenerated singlet oxygen or other ROS. Thus, Ga^3+^CHP appeared to localize more closely with aPDI-sensitive targets inside the bacterial cells, leading to higher photodynamic efficacy.

Host cell safety is critical when exploring new potential therapeutic agents. The phototherapies presented here had acceptable (Ga^3+^MPIX) or favorable (Ga^3+^CHP) safety in vitro when applied to human keratinocytes both with normal and silenced *FLG* expression. However, we observed that Ga^3+^CHP-mediated aPDI was less toxic to keratinocytes (Figure 9D) than Ga^3+^MPIX-mediated aPDI (Figure 9C), as measured by the delay in the proliferation of cells. There may be a difference in the accumulation rate inside eukaryotic cells between these two PSs, which might correspond to the greater toxicity. An important factor in aPDI and its potential use in skin decontamination is exposure time. In PDT, light delivery should last for a few seconds or minutes and ensure effective PS excitation. Green light doses were lower for Ga^3+^CHP than for Ga^3+^MPIX, which in practice corresponds to shorter irradiation times (∼10–20 min for Ga^3+^CHP; 50 min for Ga^3+^MPIX, 10.6 mW/cm^2^). It was previously reported that only 10 s of blue light irradiation (405 nm, 140 mW/cm^2^) was sufficient to eliminate >6 log_10_ viable numbers of *S. aureus* using Ga^3+^PPIX. However, this porphyrin excitation was performed in the Soret band, which is the spectrum with the highest absorption coefficient. As a result, a shorter time is sufficient for providing the optimal energy dose to excite the compound. However, blue light treatment might be mildly cytotoxic to eukaryotic cells.^54^ Excitation with the green Q band presented in this paper was very effective in activating Ga^3+^MPIX or Ga^3+^CHP while being safe for eukaryotic cells. Moreover, we did not observe mutagenic effects (Figure 8).

The main cellular target of aPDI treatment is cellular proteins. Other biomolecules, such as lipids, sugars, or DNA, may also be targeted by aPDI depending on the type of PS and its location. Nevertheless, proteins are the main target of photogenerated ROS attacks, mainly because they are the most abundant biomolecules. Additionally, virulence factors such as the superantigens presented in this work, which are extremely resistant to physical factors (heat, proteolysis, acidic environment, and desiccation), can be effectively inactivated by photogenerated ROS in vitro (Figures 10G,H and 11C,D). aPDI might be a potential method for the inactivation of bacterial virulence factors. Our team reported that aPDI treatment reduced the activity of exogenous virulence factors in *S. aureus*.^55^ Additionally, other groups showed the effect of aPDI (based on 665 nm laser light combined with methylene blue) on the biological activity of V8 protease, α-hemolysin, and sphingomyelinase produced by *S. aureus*.^56^ White light-activated Tetra-Py^+^-Me decreased the activity level of staphylococcal enterotoxin A (SEA) and SEC toxins by approximately 68% according to a reverse passive latex agglutination (RPLA) test.^57^ These results demonstrated that aPDI can effectively inactivate virulence factors in vitro, but in vivo verification of this feature is required to demonstrate the biological relevance of the aPDI-based approach. In our analyses, we used a very sensitive assay that measures the superantigen activity of the tested toxins, namely, measurement of IL-2 produced by toxin-exposed T lymphocytes. SEC and TSST-1 are potent, nonspecific superantigens for T cells (stimulating over 50% of the T cell pool) that bind to the T-cell receptor β-chain (TCR-Vβ) region or major histocompatibility complex (MHC) class II molecules.^58^ Superantigens are inducers of proinflammatory cytokines, which promote and exacerbate skin inflammation in AD patients.^59^ The production of enterotoxins by *S. aureus* is correlated with a more severe course of AD.^29^ However, animal model studies, such as in vivo mouse model studies, should be conducted to verify whether aPDI might impact the biological function of SEs.

We hypothesized that singlet oxygen could cause oxidative damage at the toxin-binding site, possibly diminishing superantigenicity. A previous report showed that ROS production led to the modification of prosthetic groups, modification of amino acid residues, fragmentation, crosslinking, and protein aggregation.^60^ Oxidation may also alter the structure of the cysteine SE loop, which is thought to be responsible for its emetic activity, and the dodecapeptide region, which is responsible for epithelial penetration of TSST-1 in menstrual toxic shock syndrome (TSS).

Another important aspect of aPDI action in the cell is the change in the level of gene expression. Published data show that aPDI downregulates the expression of genes related to biofilm production and virulence factors in several microbial species.^61,62^ Staphylococcal enterotoxin *seb* gene expression was significantly downregulated after RB- or new methylene blue-mediated aPDI.^27^ Our experiments indicated a decrease in *sec* expression levels under the influence of aPDI, which is pronounced in the case of Ga^3+^CHP, while in the case of Ga^3+^MPIX, the action of the compound itself cannot be distinguished from aPDI. In contrast, the analysis of *tst* expression levels unexpectedly showed a significant increase under the influence of aPDI with Ga^3+^CHP but not with Ga^3+^MPIX. This result is difficult to interpret and most likely depends on the differences in the properties of both compared PSs as well as aPDI protocols (optimal for each PS but different in terms of concentration 1 μM Ga^3+^CHP vs 10 μM Ga^3+^MPIX and light dose—1.59 vs 12.7 J/cm^2^). The differential expression of both genes may have also resulted from the different regulatory pathways of the individual toxins.^63^ For instance, the alternative sigma factor σB is involved in the upregulation of the *tst* gene and the downregulation of the *seb* gene.^64^ The activity of σB can be altered by several environmental factors, one of which is aPDI.^65^ Likewise, the two-component SrrAB system senses the transition from respiratory to nonrespiratory growth conditions and regulates the expression of virulence factors such as TSST-1.^33^ In aerobic growth, SrrAB upregulates the transcription of the *tst* gene, while in anaerobic growth, there is a significant downregulation of the *tst* gene.^66^ SrrAB expression may be altered by the production of singlet oxygen during aPDI (Figure 12). Such an alteration in this regulatory system has thus far been documented only after exposure to H_2_O_2_ and hypoxia. The Δ*srrAB* mutant was shown to be sensitive to H_2_O_2_ exposure and to decrease the expression levels of genes involved in H_2_O_2_ resistance. The SrrAB system regulates the transcription of both virulence factor genes and genes involved in aerobic respiration and H_2_O_2_ resistance.^33,66^ Depending on the state of the respiratory system, *S. aureus* can modify its virulence. Therefore, studying the regulation of gene expression, especially from the point of view of microorganism pathogenicity, in response to photooxidative stress is extremely important for evaluating the safety of the aPDI method.

## 5 Conclusions

This study showed the success of aPDI treatment with Ga^3+^MPs in the decolonization of clinical *S. aureus* isolates in planktonic cultures and in an ex vivo porcine skin model. The novel Ga^3+^MP, Ga^3+^CHP, activated with green light effectively reduced the survival of clinical *S. aureus* isolates derived from AD patients and in aPDI treatment of HaCaT keratinocytes with both normal and suppressed filaggrin expression. In addition, the test compound did not show mutagenic activity. Ga^3+^CHP is an efficient generator of singlet oxygen and can be recognized by cellular heme transport systems, mainly Isd, which underlies the efficient accumulation of this compound in bacterial cells. The Ga^3+^CHP photodynamic method eliminates the biological activity of the SEC or TSST-1 superantigens, which are clinically relevant *S. aureus* virulence factors. Green light-activated Ga^3+^MPs (Ga^3+^CHP-mediated aPDI) may be a potential therapeutic strategy in the decolonization of *S. aureus* on atopic skin.

## Supporting Information Available

Heat generation during irradiation; accumulation of Ga^3+^MPIX and Ga^3+^CHP; effect of Ga^3+^CHP and Ga^3+^MPIX aPDI on the *S. aureus* biofilm; evaluation of bacterial viability of *S. aureus* XEN40 on an ex vivo porcine skin model; cyto- and phototoxicity of gallium compounds on Ames assay indicator strains; and qRT-PCR conditions used in this study (PDF)
