# Supplementary material for: Photoactivated Gallium Porphyrin Reduces Staphylococcus aureus Colonization on the Skin and Suppresses Its Ability to Produce Enterotoxin C and TSST-1
Source: Mol Pharm. 2023 Sep 1;20(10):5108–24. doi: 10.1021/acs.molpharmaceut.3c00399 (PMC10553792; doi:10.1021/acs.molpharmaceut.3c00399)
Supplement: Supplementary file 1 — mp3c00399_si_001.pdf [file mp3c00399_si_001.pdf]

Photoactivated gallium porphyrin reduces *Staphylococcus aureus* colonization on the skin and suppresses its ability to produce enterotoxin C and TSST-1

Klaudia Szymczak, Grzegorz Szewczyk, Michał Rychłowski, Tadeusz Sarna, Lei Zhang, Mariusz Grinholc, Joanna Nakonieczna (#)

- Supplementary Materials

1. **Heat generation during irradiation** 2
2. **Accumulation of Ga^{3+}MPIX and Ga^{3+}CHP** 3
3. **Effect of Ga^{3+}CHP and Ga^{3+}MPIX aPDI on *S. aureus* biofilm** 4
4. **Evaluation of bacterial viability of *S. aureus* XEN40 on ex vivo porcine skin model** 5
5. **The cyto- and phototoxicity of gallium compounds on Ames assay indicator strains** 6
6. **Supplementary information on qRT-PCR** 7

1. Heat generation during irradiation

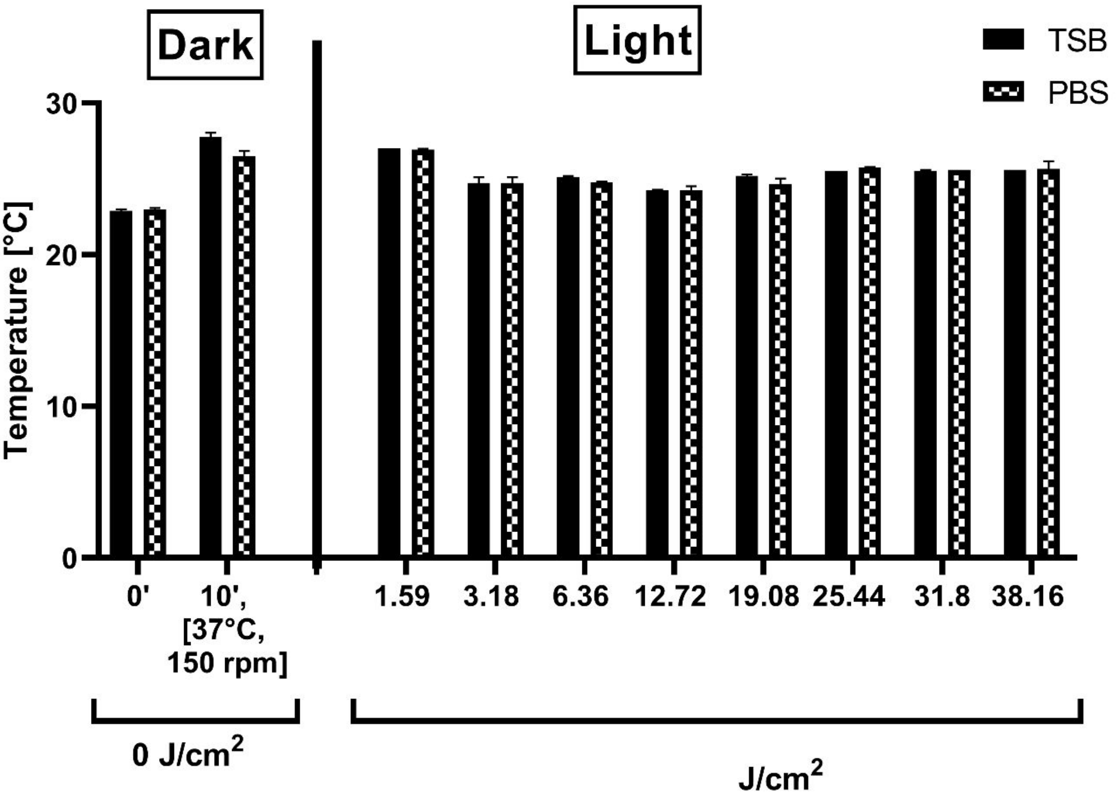

Figure S1. Temperature measurement over time. Bacterial cells were incubated in Tryptic soy broth (TSB) medium or phosphate buffer saline (PBS) for 10 minutes in the dark at 37 °C (Dark). The cells were then exposed to light (LED, 10.6 mW/cm^2) at 522 nm for up to 60 min. Temperature was measured at successive time points corresponding to specific light doses (indicated on the X axis).

2. Accumulation of Ga^{3+}MPIX and Ga^{3+}CHP

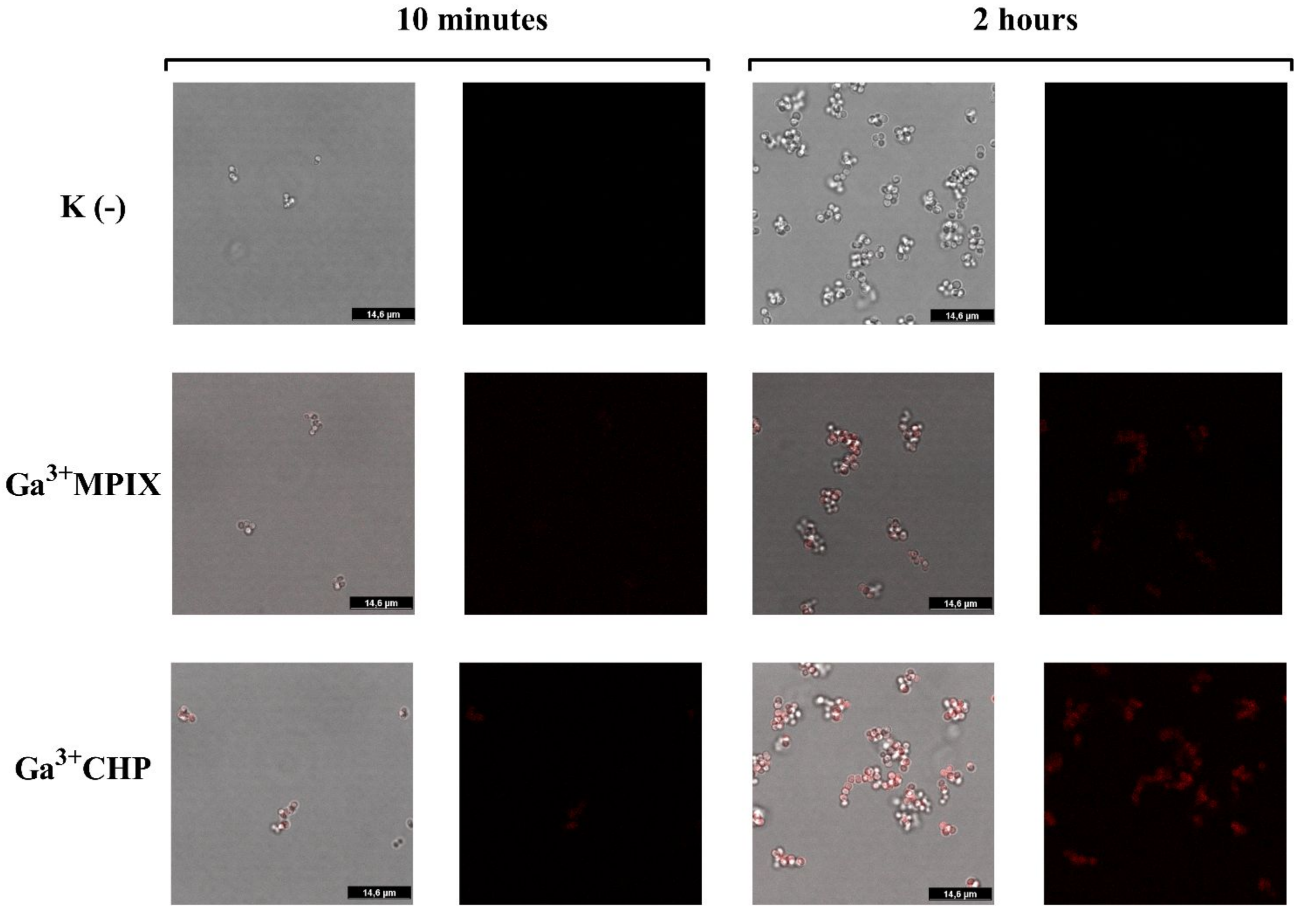

Figure S2. Ga^{3+}MPIX and Ga^{3+}CHP uptake in S. *aureus* 25923. Overnight bacterial cultures were diluted and incubated with 10 µM of each photosensitizer for 10 minutes or 2 hours at 37°C with shaking. Then, washed once with PBS buffer. Specimens were imaged with a Leica SP8X confocal laser scanning microscope with a 100× immersion lens with excitation at 405 nm and fluorescence emission at 551-701 nm (Leica, Germany).

3. Effect of Ga^{3+}CHP and Ga^{3+}MPIX aPDI on *S. aureus* biofilm

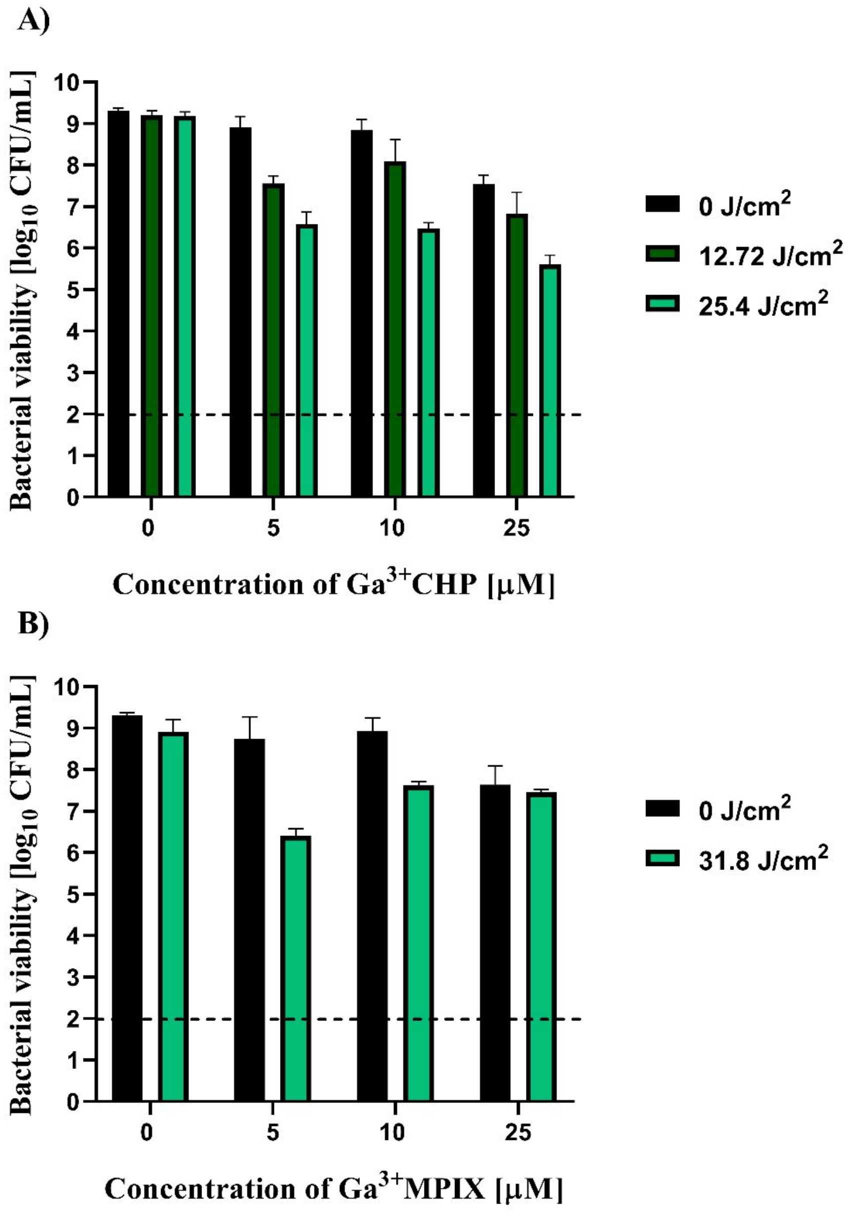

Figure S3. Effect of Ga^{3+}CHP and Ga^{3+}MPIX aPDI on *S. aureus* 5N biofilm viability. Overnight bacterial cultures were diluted to 10^7 CFU/mL and placed into 96-well microtiter plate. Then, after 4h incubation at 37°C the medium was removed, replaced with 200 µL of fresh medium, and incubated at 37 °C for 20 h. Afterward, the biofilm was washed, and then 30 minutes of incubation with photosensitizer (Ga^{3+}CHP or Ga^{3+}MPIX; 0-25 µM) started, then washed once and illuminated with 522 nm light at the doses indicated in the legend. After dispersing and serial dilutions, samples were plated on TSA plates to evaluate the impact on the biofilm viability.

4. Evaluation of bacterial viability of *S. aureus* XEN40 on ex vivo porcine skin model

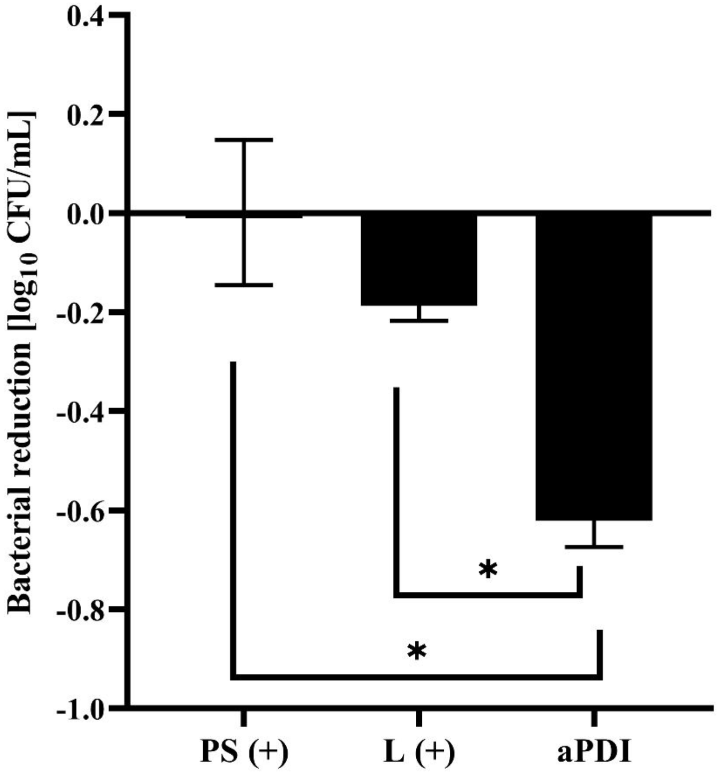

Figure S4. Evaluation of bacterial viability of *S. aureus* XEN40 on ex vivo porcine skin model after Ga^{3+}CHP-mediated aPDI treatment. *S. aureus* XEN40 strain was applied to clean porcine skin grafts 24 hours before treatment. A 200 µL of MiliQ or 10 µM of Ga^{3+}CHP was applied on the graft and incubated at 37 °C for 10 min prior to irradiation (12.72 J/cm^2). Then, bacteria were collected with a sterile swab into 300 µL PBS. Samples were centrifuged (5min x 14 000 rcf) and resuspended in 100 µL. Bacterial suspensions were serially diluted and placed into TSA agar plates for CFU counting. Results are the mean of the *S. aureus* reduction in bacterial viability after either photosensitizer-, light- or aPDI- treatment in respect to untreated cells. Significance at the respective p-values is marked with an asterisk (* $p < 0.05$) with respect to the "aPDI" group by Dunnett's multiple comparisons test.

5. The cyto- and phototoxicity of gallium compounds against indicator strains used in the Ames test

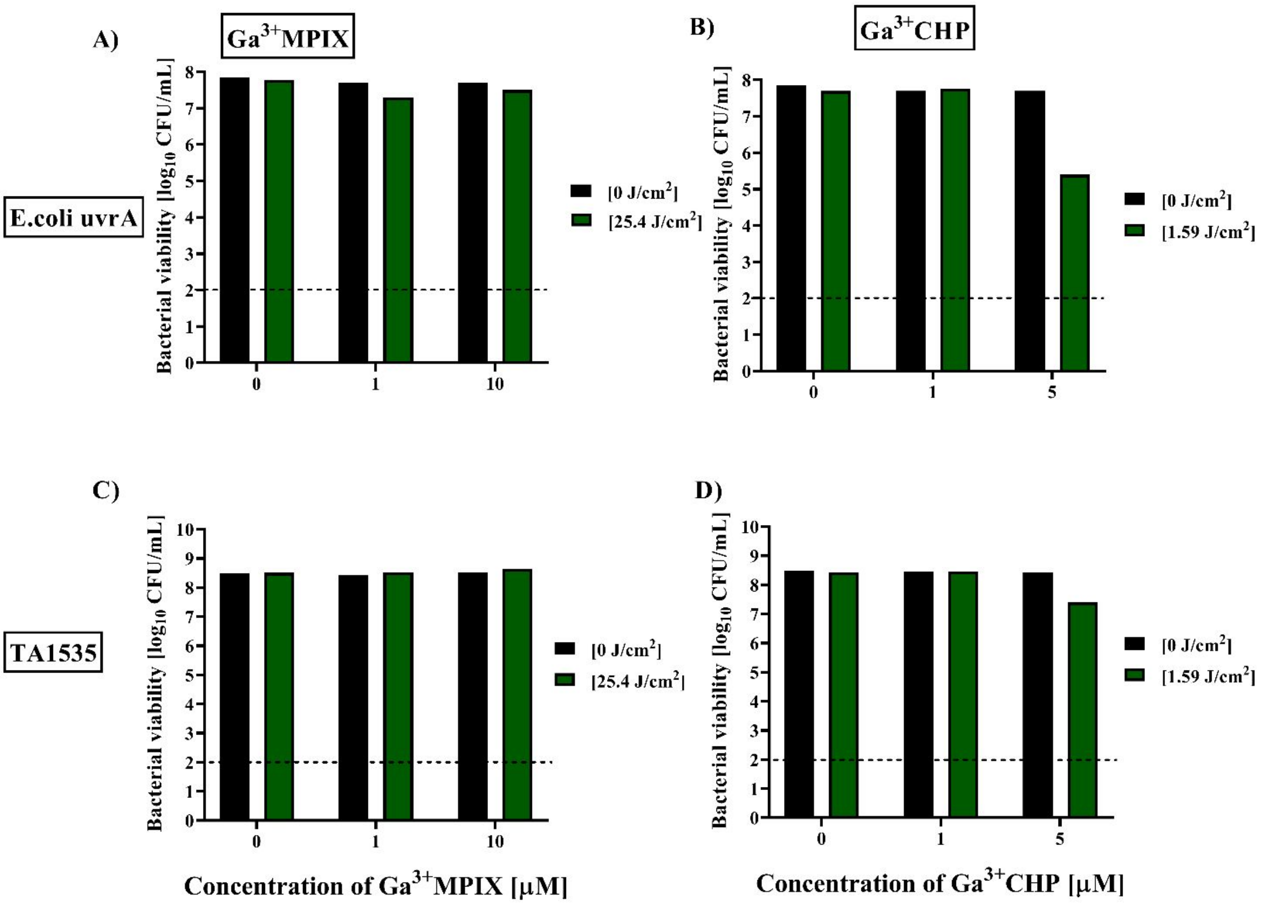

Figure S5. The cyto- and phototoxicity of gallium compounds against indicator strains (*E. coli uvrA* and TA1535). *E. coli* uvrA (A, B) and TA1535 (C, D) strains were exposed to both gallium compounds: Ga^{3+}MPIX (A, C) or Ga^{3+}CHP (B, D) either in the dark or under green light conditions. For the light-activated treatment groups, after 10 minutes of incubation with compounds, cells were exposed to the green light at the proper dosage (25.4 J/cm^2 for Ga^{3+}MPIX or 1.59 J/cm^2 for Ga^{3+}CHP). Then, cells were serially diluted and placed on the TSA agar plates to examine the bacterial viability.

6. Supplementary information on qRT-PCR

Table S1. Primers used into qRT-PCR analysis ("F-forward; "R"-Reverse)

Gene	Primer sequence (5'-3')
gmk	**F:** AATCGTTTTATCAGG ACC<br>**R:** CTTCACCTTCACGCATTT
sec	**F:** AATAAAACGGTTGATTCTAAAAGTGTGAA<br>**R:** ATCAAAATCGGATTAACATTATCCATTC
tst	**F:** TCATCAGCTAACTCAAATACATGGATT<br>**R:** TGTGGATCCGTCATTCATTGTT
srrA	**F:** AGCATGTGTGGGAGGTATGA<br>**R:** CCTCTTGGCCATTACTTGCTT
srrB	**F:**AGCCGGCTAAATAGTGTCGT<br>**R:**ATGGCATTTTCGGTTTCTTG

Table S2. qRT-PCR conditions used in this study.

Step	Temperature	Time	Cycles
Pre-incubation	95 °C	5 min	1
Amplification	95 °C	15 s	45
	60 °C	15 s	
	72 °C	15 s	
Melting curves	95 °C	5 s	1
	65 °C -> 97 °C	1 min	
Cool down	40 °C	30 s	1
